# Supplementary material for: The relative effectiveness of law enforcement policies aimed at reducing illegal trade: Evidence from laboratory markets
Source: PLoS One. 2021 Nov 2;16(11):e0259254. doi: 10.1371/journal.pone.0259254 (PMC8562810; doi:10.1371/journal.pone.0259254)
Supplement: S1 Text — (DOCX) [file pone.0259254.s001.docx]

# **Supporting information: S1 Text. Experimental instructions**

#

## **No Seizure treatment instructions**

***Introduction***

This is an experiment in the economics of market decision making. In this experiment, we will set up a market in which some of you will be BUYERS and some of you will be SELLERS.

The commodity you are trading is referred to as a "unit". Sellers make earnings by producing units at a cost and selling these units to buyers. Buyers make earnings by purchasing units from sellers and then redeeming (or reselling) these units to the experimenter. Earnings are recorded in a fictitious currency called tokens. Tokens are exchanged for cash at the rate of **100 tokens = $1.00**. Your earnings will be paid to you in CASH at the end of the experiment. To begin, every seller and buyer will be given an initial balance of **1500 tokens ($15.00)**. You may keep this money PLUS any you earn.

Buyers and sellers will be randomly paired and will exchange units for tokens in computerized markets over a sequence of trading periods. Each trading period consists of three one-minute bargaining rounds during which pairs of buyers and sellers negotiate trading prices.

All trading is conducted over the computer network. Each trading period will consist of a production period followed by random pairings of buyers and sellers and negotiations for trade prices. During the production period, the sellers determine the number of units to produce and the buyers wait. After the sellers have produced the units, buyers and sellers are randomly paired in each of the three one-minute bargaining rounds during which each buyer and seller pair negotiate for trade prices. See the figure below:

BUYERS WAIT

BARGAINING CONVERT TOKEN

ROUNDS EARNINGS TO

DOLLARS AND CENTS

SELLERS PRODUCE UNITS

For the units produced, the cost of production is deducted from the seller's token balance. In addition, the computer will automatically account for sales or purchases that you have made and adjust your token balance accordingly. A listing of sales or purchases you have made and your adjusted token balance will be displayed on the computer screen at the end of every trading period. After you have viewed this information and clicked on OK, a new trading period with three trading rounds will begin. This experiment will consist of several trading periods. We will conduct a practice trading period to familiarize you with the mechanics of the computerized market before the actual experiment begins. During the practice trading period the information you see will be different than that in the actual experiment.

***Specific Instructions to Buyers***

During each trading period you are free to purchase up to 8 units. For the first unit that you buy during a trading period, you will receive the amount listed under UNIT VALUE for Unit 1. In this example, this amount is 55 tokens. For the second unit that you buy you will receive the amount listed under UNIT VALUE for Unit 2, which is 50 tokens. The redemption values for these and subsequent units will be displayed on your computer screen.

The earnings from each unit that you purchase (which are yours to keep) are computed by taking the difference between the redemption value and purchase price of the unit bought. That is,

**Your Earnings = Redemption Value - Purchase Price.**

Suppose, for example, that you buy 2 units in a trading period. If you pay 40 tokens for the first unit and 30 tokens for the second unit your earnings are:

**earnings for Unit 1 = 55 - 40 = 15**

**earnings for Unit 2 = 50 - 30 = 20.**

Your total earnings are:

**total earnings = 15 + 20 = 35 tokens.**

During the experiment this trading information will be summarized on the computer screen at the end of each trading period. Buyers also should be aware that they will not be allowed to spend more tokens buying units than what they have in their beginning balance in any one period.

***Specific Instructions to Sellers***

During each trading period you are free to sell up to 8 units. Remember, you must decide on the number of units you wish to produce and then sell in the production period.

The first unit that you produce during a trading period will cost you the amount listed under UNIT COST for Unit 1. In this example, this cost is 15 tokens. Unit 1's unit cost is 15 tokens. The second unit that you produce will cost you the amount listed under UNIT COST for Unit 2, which is 20 tokens and Unit 3 is 25 tokens. The unit costs for these and subsequent units will be displayed on your computer screens.

The earnings from each unit that you sell (which are yours to keep) are computed by taking the difference between the sale price and unit cost of the unit sold. That is,

**Your Earnings = Sale Price - Unit Cost**

You sell Unit 1 for 45 tokens, Unit 2 for 40 tokens and Unit 3 for 35 tokens. Your earnings would then be:

**earnings for Unit 1 = 45 - 15 = 30**

**earnings for Unit 2 = 40 - 20 = 20**

**earnings for Unit 3 = 35 - 25 = 10**

Your total earnings are:

**total earnings = 30 + 20 + 10 = 60 tokens**

During the experiment this trading information will be summarized on the computer screen at the end of each trading period. Sellers also should be aware that they will not be allowed to incur a production cost greater than the amount in their beginning token balance in any one period.

***Trading Rules for the Market***

Only one unit may be bought and sold at a time. A buyer makes bids to the seller to purchase a unit. A "bid" is a proposed price at which a buyer is willing to purchase a unit. Bids must become progressively higher. In other words, if the first bid for a unit is 50 tokens, then the second bid must be higher than 50. Suppose the second bid is 55 tokens, then the third bid must be higher than 55, and so on.

A seller makes offers to the buyer to sell a unit. An "offer" is a proposed price at which a seller is willing to sell a unit. Offers must become progressively lower. In other words, if the first offer to sell a unit is for 60 tokens, then the second offer must be lower than 60. Suppose the second offer is 55 tokens, then the third offer must be less than 55, and so on.

There is one further set of restrictions on bids and offers. The reason for these restrictions is just common sense. A buyer's bid cannot be higher than what is labeled on the computer screen as the BEST OFFER. In other words, a buyer cannot attempt to pay a price that is higher than that for which the seller is willing to sell. Similarly, a seller's offer cannot be lower than what is labeled as the BEST BID. In other words, a seller cannot attempt to sell at a price below that which the buyer is willing to pay. In fact, the computer will not allow such bids and offers.

After a trade has been made, bid and offer values are cleared from the screen. A buyer and seller pair may then resume entering bids and offers for additional units. Trades are made between buyer and seller pairs for one minute. After a minute has elapsed, buyers and sellers are again randomly paired and the next trading round begins.

Each trading period has a maximum time limit of 3 minutes or three one-minute trading rounds. A trading period will be terminated automatically if profitable trades cannot be made by the randomly matched buyer and seller. Because buyers incur a purchase price for units and sellers incur costs to produce units for sale, it is possible to reach a token balance of 0. If a player reaches a 0 token balance, they cannot continue to buy or produce units in the market and the experimental session will end. There will be at least 20 periods in the experiment. There is a 1 in 5 chance that period 20 will be the last period. For every period after 20, the probability of a random stop is 1 in 5.

***Your Name and ID Number***

Before the practice trading period, the computer will ask for your name and ID number. This information is kept confidential, but it is important to the funding agency as proof of your participation. The bids and earnings of people in the experiment are confidential. Please do not look at someone else’s screen and do not speak to another participant once the experiment begins. You may ask the experimenter questions at any time during the experiment. Are there any questions before we conduct the practice trading period?

## **Seller Profit Seizure treatment instructions**

***Introduction***

This is an experiment in the economics of market decision making. In this experiment, we will set up a market in which some of you will be BUYERS and some of you will be SELLERS.

The commodity you are trading is referred to as a "unit". Sellers make earnings by producing units at a cost and selling these units to buyers. Buyers make earnings by purchasing units from sellers and then redeeming (or reselling) these units to the experimenter. Earnings are recorded in a fictitious currency called tokens. Tokens have a cash value of **100 tokens = $1.00.** Your earnings will be paid to you in CASH at the end of the experiment. To begin, every seller and buyer will be given an initial balance of **1500 tokens ($15.00).** You may keep this money PLUS any you earn.

Buyers and sellers will be randomly paired and will exchange units for tokens in computerized markets over a sequence of trading periods. Each trading period consists of three one-minute bargaining rounds during which pairs of buyers and sellers negotiate trading prices. All trading is conducted over the computer network. Each trading period will consist of a production period followed by random pairings of buyers and sellers and negotiations for trade prices. During the production period, the sellers determine the number of units to produce and the buyers wait. After the sellers have produced the units, buyers and sellers are randomly paired in each of the three one-minute bargaining rounds during which each buyer and seller pair negotiate for trade prices. See the figure below:

BUYERS WAIT

BARGAINING CONVERT TOKEN

ROUNDS EARNINGS TO

DOLLARS AND CENTS

SELLERS PRODUCE UNITS

or the units produced, the cost of production is deducted from the seller's token balance. In addition, the computer will automatically account for sales or purchases that you have made and adjust your token balance accordingly. A listing of sales or purchases you have made and your adjusted token balance will be displayed on the computer screen at the end of every trading period.

After you have viewed this information and clicked on OK, a new trading period with three trading rounds will begin. This experiment will consist of several trading periods. We will conduct a practice trading period to familiarize you with the mechanics of the computerized market before the actual experiment begins. During the practice trading period the information you see will be different than that in the actual experiment.

***Specific Instruction to Buyers***

During each trading period you are free to purchase up to 8 units. For the first unit that you buy during a trading period, you will receive the amount listed under UNIT VALUE for Unit 1. In this example, this amount is 55 tokens. For the second unit that you buy you will receive the amount listed under UNIT VALUE for Unit 2, which is 50 tokens. The redemption values for these and subsequent units will be displayed on your computer screen.

The earnings from each unit that you purchase (which are yours to keep) are computed by taking the difference between the redemption value and purchase price of the unit bought. That is,

**Your Earnings = Redemption Value - Purchase Price.**

Suppose, for example, that you buy 2 units in a trading period. If you pay 40 tokens for the first unit and 30 tokens for the second unit your earnings are:

**earnings for Unit 1 = 55 - 40 = 15**

**earnings for Unit 2 = 50 - 30 = 20.**

Your total earnings are:

**total earnings = 15 + 20 = 35 tokens.**

During the experiment this trading information will be summarized on the computer

screen at the end of each trading period. Tokens will be converted to cash at the conversion rate of 100 tokens = $1.00. Buyers also should be aware that they will not be allowed to spend more tokens buying units than what they have in their beginning balance in any one period.

***Specific Instructions to Sellers***

You must decide on the number of units you wish to produce and then sell in the production period. During each trading period you are free to sell up to 8 units. If a trade is not completed, you incur production costs but do not sell the unit or receive the sale price. Because you must decide on the number of units to produce before you sell them in the production period, you will also incur production costs for all units you produce, even the ones you do not sell in the trading period. The first unit that you sell during a trading period will cost you the amount listed under UNIT COST for Unit 1. In this example, this cost is 15 tokens. Unit 1's unit cost is 15 tokens. The second unit that you sell will cost you the amount listed under UNIT COST for Unit 2, which is 20 tokens and Unit 3 is 25 tokens. The unit costs for these and subsequent units will be displayed on your computer screens.

The earnings from each unit that you sell (which are yours to keep) are computed by

taking the difference between the sale price and unit cost of the unit sold. That is,

**Your Earnings = Sale Price - Unit Cost**

Let's suppose that in the trading period you sell Unit 1 for 45 tokens, Unit 2 for 40 tokens and Unit 3 for 35 tokens. Your earnings would then be:

**earnings for Unit 1= 45 - 15 = 30**

**earnings for Unit 2 = 40 - 20 = 20**

**earnings for Unit 3 = 35 - 25 = 10**

Your total earnings are:

**total earnings = 30 + 20 + 10 = 60 tokens**

During the experiment this trading information will be summarized on the computer screen at the end of each trading period. Sellers also should be aware that they will not be allowed to incur a production cost greater than the amount in their beginning token balance in any one period.

**Also for sellers, when tokens are converted to dollars at the end of a trading period as shown in the above figure, there is a conversion fee that will range between zero and all of your token earnings for the trading period. The average loss is 20% of your token earnings. Hence the conversion fee is random each period. The computer screen will convert tokens to dollars at the end of each trading period and show you the conversion fee levied.**

***Trading Rules for the Market***

Only one unit may be bought and sold at a time. A buyer makes bids to the seller to purchase a unit. A "bid" is a proposed price at which a buyer is willing to purchase a unit. Bids must become progressively higher. In other words, if the first bid for a unit is 50 tokens, then the second bid must be higher than 50. Suppose the second bid is 55 tokens, then the third bid must be higher than 55, and so on.

A seller makes offers to the buyer to sell a unit. An "offer" is a proposed price at which a seller is willing to sell a unit. Offers must become progressively lower. In other words, if the first offer to sell a unit is for 60 tokens, then the second offer must be lower than 60. Suppose

the second offer is 55 tokens, then the third offer must be less than 55, and so on.

There is one further set of restrictions on bids and offers. The reason for these restrictions is just common sense. A buyer's bid cannot be higher than what is labeled on the

computer screen as the BEST OFFER. In other words, a buyer cannot attempt to pay a price that is higher than that for which the seller is willing to sell. Similarly, a seller's offer cannot be lower than what is labeled as the BEST BID. In other words, a seller cannot attempt to sell at a

price below that which the buyer is willing to pay. In fact, the computer will not allow such bids and offers.

After a seller and buyer have made a trade, the trading price will be listed on both the buyer's and seller's screens. After a trade has been made, bid and offer values are cleared from the screen. A buyer and seller pair may then resume entering bids and offers for additional units. Trades are made between buyer and seller pairs for one minute. After a minute has elapsed, buyers and sellers are again randomly paired and the next trading round begins.

Each trading period has a maximum time limit of 3 minutes or three one-minute trading rounds. A trading period will be terminated automatically if profitable trades cannot be made by the randomly matched buyer and seller. Because buyers incur a purchase price for units and sellers incur costs to produce units for sale, it is possible to reach a token balance of 0. Ifa player reaches a 0 token balance, they cannot continue to buy or produce units in the market and the experimental session will end. There will be at least 20 periods in the experiment. There is a 1 in 5 chance that period 20 will be the last period. For every period after 20, the probability of a random stop is 1 in 5.

***Your Name and ID Number***

Before the practice trading period, the computer will ask for your name and ID number. This information is kept confidential, but it is important to the funding agency as proof of your participation. The bids and earnings of people in the experiment are confidential. Please do not look at someone else's screen and do not speak to another participant once the experiment begins. You may ask the experimenter questions at any time during the experiment. Are there any questions before we conduct the practice trading period?

**Buyer Profit Seizure treatment instructions**

***Introduction***

This is an experiment in the economics of market decision making. In this experiment, we will set up a market in which some of you will be BUYERS and some of you will be SELLERS.

The commodity you are trading is referred to as a "unit". Sellers make earnings by producing units at a cost and selling these units to buyers. Buyers make earnings by purchasing units from sellers and then redeeming (or reselling) these units to the experimenter. Earnings are recorded in a fictitious currency called tokens. Tokens have a cash value of **100 tokens = $1.00.** Your earnings will be paid to you in CASH at the end of the experiment. To begin, every seller and buyer will be given an initial balance of **1500 tokens ($15.00).** You may keep this money PLUS any you earn.

Buyers and sellers will be randomly paired and will exchange units for tokens in computerized markets over a sequence of trading periods. Each trading period consists of three one-minute bargaining rounds during which pairs of buyers and sellers negotiate trading prices. All trading is conducted over the computer network. Each trading period will consist of a production period followed by random pairings of buyers and sellers and negotiations for trade prices. During the production period, the sellers determine the number of units to produce and the buyers wait. After the sellers have produced the units, buyers and sellers are randomly paired in each of the three one-minute bargaining rounds during which each buyer and seller pair negotiate for trade prices. See the figure below:

BUYERS WAIT

BARGAINING CONVERT TOKEN

ROUNDS EARNINGS TO

DOLLARS AND CENTS

SELLERS PRODUCE UNITS

or the units produced, the cost of production is deducted from the seller's token balance. In addition, the computer will automatically account for sales or purchases that you have made and adjust your token balance accordingly. A listing of sales or purchases you have made and your adjusted token balance will be displayed on the computer screen at the end of every trading period.

After you have viewed this information and clicked on OK, a new trading period with three trading rounds will begin. This experiment will consist of several trading periods. We will conduct a practice trading period to familiarize you with the mechanics of the computerized market before the actual experiment begins. During the practice trading period the information you see will be different than that in the actual experiment.

***Specific Instructions to Buyers***

During each trading period you are free to purchase up to 8 units. For the first unit that you buy during a trading period, you will receive the amount listed under UNIT VALUE for Unit 1. In this example, this amount is 55 tokens. For the second unit that you buy you will receive the amount listed under UNIT VALUE for Unit 2, which is 50 tokens. The redemption values for these and subsequent units will be displayed on your computer screen.

The earnings from each unit that you purchase (which are yours to keep) are computed by taking the difference between the redemption value and purchase price of the unit bought. That

is,

**Your Earnings = Redemption Value - Purchase Price**

Suppose, for example, that you buy 2 units in a trading period. If you pay 40 tokens for the first unit and 30 tokens for the second unit your earnings are:

**earnings for Unit 1= 55 - 40 = 15**

**earnings for Unit 2 = 50 - 30 = 20**

Your total earnings are:

**total earnings = 15 + 20 = 35 tokens**

During the experiment this trading information will be summarized on the computer

screen at the end of each trading period. Tokens will be converted to cash at the conversion rate of 100 tokens = $1.00. Buyers also should be aware that they will not be allowed to spend more tokens buying units than what they have in their beginning balance in any one period.

**Also for buyers, when tokens are converted to dollars at the end of a trading period as shown in the above figure, there is a conversion fee that will range between zero and all of your token earnings for the trading period. The average loss is 20% of your token earnings. Hence the conversion fee is random each period. The computer screen will convert tokens to dollars at the end of each trading period and show you the conversion fee levied.**

***Specific Instructions to Sellers***

You must decide on the number of units you wish to produce and then sell in the production period. During each trading period you are free to sell up to 8 units. If a trade is not completed, you incur production costs but do not sell the unit or receive the sale price. Because you must decide on the number of units to produce before you sell them in the production period, you will also incur production costs for all units you produce, even the ones you do not sell in the trading period. The first unit that you sell during a trading period will cost you the amount listed under UNIT COST for Unit 1. In this example, this cost is 15 tokens. Unit 1's unit cost is 15 tokens. The second unit that you sell will cost you the amount listed under UNIT COST for Unit 2, which is 20 tokens and Unit 3 is 25 tokens. The unit costs for these and subsequent units will be displayed on your computer screens.

The earnings from each unit that you sell (which are yours to keep) are computed by

taking the difference between the sale price and unit cost of the unit sold. That is,

**Your Earnings = Sale Price - Unit Cost**

Let's suppose that in the trading period you sell Unit 1 for 45 tokens, Unit 2 for 40 tokens and Unit 3 for 35 tokens. Your earnings would then be:

**earnings for Unit 1= 45 - 15 = 30**

**earnings for Unit 2 = 40 - 20 = 20**

**earnings for Unit 3 = 35 - 25 = 10**

Your total earnings are:

**total earnings = 30 + 20 + 10 = 60 tokens**

During the experiment this trading information will be summarized on the computer screen at the end of each trading period. Sellers also should be aware that they will not be allowed to incur a production cost greater than the amount in their beginning token balance in any one period.

***Trading Rules for the Market***

Only one unit may be bought and sold at a time. A buyer makes bids to the seller to purchase a unit. A "bid" is a proposed price at which a buyer is willing to purchase a unit. Bids must become progressively higher. In other words, if the first bid for a unit is 50 tokens, then the second bid must be higher than 50. Suppose the second bid is 55 tokens, then the third bid must be higher than 55, and so on.

A seller makes offers to the buyer to sell a unit. An "offer" is a proposed price at which a seller is willing to sell a unit. Offers must become progressively lower. In other words, if the first offer to sell a unit is for 60 tokens, then the second offer must be lower than 60. Suppose

the second offer is 55 tokens, then the third offer must be less than 55, and so on.

There is one further set of restrictions on bids and offers. The reason for these restrictions is just common sense. A buyer's bid cannot be higher than what is labeled on the

computer screen as the BEST OFFER. In other words, a buyer cannot attempt to pay a price that is higher than that for which the seller is willing to sell. Similarly, a seller's offer cannot be lower than what is labeled as the BEST BID. In other words, a seller cannot attempt to sell at a

price below that which the buyer is willing to pay. In fact, the computer will not allow such bids and offers.

After a seller and buyer have made a trade, the trading price will be listed on both the buyer's and seller's screens. After a trade has been made, bid and offer values are cleared from the screen. A buyer and seller pair may then resume entering bids and offers for additional units. Trades are made between buyer and seller pairs for one minute. After a minute has elapsed, buyers and sellers are again randomly paired and the next trading round begins.

Each trading period has a maximum time limit of 3 minutes or three one-minute trading rounds. A trading period will be terminated automatically if profitable trades cannot be made by the randomly matched buyer and seller. Because buyers incur a purchase price for units and sellers incur costs to produce units for sale, it is possible to reach a token balance of 0. If a player reaches a 0 token balance, they cannot continue to buy or produce units in the market and the experimental session will end. There will be at least 20 periods in the experiment. There is a 1 in 5 chance that period 20 will be the last period. For every period after 20, the probability of a random stop is 1 in 5.

***Your Name and ID Number***

Before the practice trading period, the computer will ask for your name and ID number. This information is kept confidential, but it is important to the funding agency as proof of your participation. The bids and earnings of people in the experiment are confidential. Please do not look at someone else's screen and do not speak to another participant once the experiment begins. You may ask the experimenter questions at any time during the experiment. Are there any questions before we conduct the practice trading period?

## **Both Profit Seizure treatment instructions**

***Introduction***

This is an experiment in the economics of market decision making. In this experiment, we will set up a market in which some of you will be BUYERS and some of you will be SELLERS.

The commodity you are trading is referred to as a "unit". Sellers make earnings by producing units at a cost and selling these units to buyers. Buyers make earnings by purchasing units from sellers and then redeeming (or reselling) these units to the experimenter. Earnings are recorded in a fictitious currency called tokens. Tokens have a cash value of **100 tokens = $1.00.** Your earnings will be paid to you in CASH at the end of the experiment. To begin, every seller and buyer will be given an initial balance of **1500 tokens ($15.00).** You may keep this money PLUS any you earn.

Buyers and sellers will be randomly paired and will exchange units for tokens in computerized markets over a sequence of trading periods. Each trading period consists of three one-minute bargaining rounds during which pairs of buyers and sellers negotiate trading prices. All trading is conducted over the computer network. Each trading period will consist of a production period followed by random pairings of buyers and sellers and negotiations for trade prices. During the production period, the sellers determine the number of units to produce and the buyers wait. After the sellers have produced the units, buyers and sellers are randomly paired in each of the three one-minute bargaining rounds during which each buyer and seller pair negotiate for trade prices. See the figure below:

BUYERS WAIT

BARGAINING CONVERT TOKEN

ROUNDS EARNINGS TO

DOLLARS AND CENTS

SELLERS PRODUCE UNITS

or the units produced, the cost of production is deducted from the seller's token balance. In addition, the computer will automatically account for sales or purchases that you have made and adjust your token balance accordingly. A listing of sales or purchases you have made and your adjusted token balance will be displayed on the computer screen at the end of every trading period.

After you have viewed this information and clicked on OK, a new trading period with three trading rounds will begin. This experiment will consist of several trading periods. We will conduct a practice trading period to familiarize you with the mechanics of the computerized market before the actual experiment begins. During the practice trading period the information you see will be different than that in the actual experiment.

***Specific Instructions to Buyers***

During each trading period you are free to purchase up to 8 units. For the first unit that you buy during a trading period, you will receive the amount listed under UNIT VALUE for Unit 1. In this example, this amount is 55 tokens. For the second unit that you buy you will receive the amount listed under UNIT VALUE for Unit 2, which is 50 tokens. The redemption values for these and subsequent units will be displayed on your computer screen.

The earnings from each unit that you purchase (which are yours to keep) are computed by taking the difference between the redemption value and purchase price of the unit bought. That

is,

**Your Earnings = Redemption Value - Purchase Price**

Suppose, for example, that you buy 2 units in a trading period. If you pay 40 tokens for the first unit and 30 tokens for the second unit your earnings are:

**earnings for Unit 1= 55 - 40 = 15**

**earnings for Unit 2 = 50 - 30 = 20**

Your total earnings are:

**total earnings = 15 + 20 = 35 tokens**

During the experiment this trading information will be summarized on the computer

screen at the end of each trading period. Tokens will be converted to cash at the conversion rate of 100 tokens = $1.00. Buyers also should be aware that they will not be allowed to spend more tokens buying units than what they have in their beginning balance in any one period.

**Also for buyers, when tokens are converted to dollars at the end of a trading period as shown in the above figure, there is a conversion fee that will range between zero and all of your token earnings for the trading period. The average loss is 20% of your token earnings. Hence the conversion fee is random each period. The computer screen will convert tokens to dollars at the end of each trading period and show you the conversion fee levied.**

***Specific Instructions to Sellers***

You must decide on the number of units you wish to produce and then sell in the production period. During each trading period you are free to sell up to 8 units. If a trade is not completed, you incur production costs but do not sell the unit or receive the sale price. Because you must decide on the number of units to produce before you sell them in the production period, you will also incur production costs for all units you produce, even the ones you do not sell in the trading period. The first unit that you sell during a trading period will cost you the amount listed under UNIT COST for Unit 1. In this example, this cost is 15 tokens. Unit 1's unit cost is 15 tokens. The second unit that you sell will cost you the amount listed under UNIT COST for Unit 2, which is 20 tokens and Unit 3 is 25 tokens. The unit costs for these and subsequent units will be displayed on your computer screens.

The earnings from each unit that you sell (which are yours to keep) are computed by

taking the difference between the sale price and unit cost of the unit sold. That is,

**Your Earnings = Sale Price - Unit Cost**

Let's suppose that in the trading period you sell Unit 1 for 45 tokens, Unit 2 for 40 tokens and Unit 3 for 35 tokens. Your earnings would then be:

**earnings for Unit 1= 45 - 15 = 30**

**earnings for Unit 2 = 40 - 20 = 20**

**earnings for Unit 3 = 35 - 25 = 10**

Your total earnings are:

**total earnings = 30 + 20 + 10 = 60 tokens**

During the experiment this trading information will be summarized on the computer screen at the end of each trading period. Sellers also should be aware that they will not be allowed to incur a production cost greater than the amount in their beginning token balance in any one period.

**Also for sellers, when tokens are converted to dollars at the end of a trading period as shown in the above figure, there is a conversion fee that will range between zero and all of your token earnings for the trading period. The average loss is 20% of your token earnings. Hence the conversion fee is random each period. The computer screen will convert tokens to dollars at the end of each trading period and show you the conversion fee levied.**

***Trading Rules for the Market***

Only one unit may be bought and sold at a time. A buyer makes bids to the seller to purchase a unit. A "bid" is a proposed price at which a buyer is willing to purchase a unit. Bids must become progressively higher. In other words, if the first bid for a unit is 50 tokens, then the second bid must be higher than 50. Suppose the second bid is 55 tokens, then the third bid must be higher than 55, and so on.

A seller makes offers to the buyer to sell a unit. An "offer" is a proposed price at which a seller is willing to sell a unit. Offers must become progressively lower. In other words, if the first offer to sell a unit is for 60 tokens, then the second offer must be lower than 60. Suppose

the second offer is 55 tokens, then the third offer must be less than 55, and so on.

There is one further set of restrictions on bids and offers. The reason for these restrictions is just common sense. A buyer's bid cannot be higher than what is labeled on the

computer screen as the BEST OFFER. In other words, a buyer cannot attempt to pay a price that is higher than that for which the seller is willing to sell. Similarly, a seller's offer cannot be lower than what is labeled as the BEST BID. In other words, a seller cannot attempt to sell at a

price below that which the buyer is willing to pay. In fact, the computer will not allow such bids and offers.

After a seller and buyer have made a trade, the trading price will be listed on both the buyer's and seller's screens. After a trade has been made, bid and offer values are cleared from the screen. A buyer and seller pair may then resume entering bids and offers for additional units. Trades are made between buyer and seller pairs for one minute. After a minute has elapsed, buyers and sellers are again randomly paired and the next trading round begins.

Each trading period has a maximum time limit of 3 minutes or three one-minute trading rounds. A trading period will be terminated automatically if profitable trades cannot be made by the randomly matched buyer and seller. Because buyers incur a purchase price for units and sellers incur costs to produce units for sale, it is possible to reach a token balance of 0. Ifa player reaches a 0 token balance, they cannot continue to buy or produce units in the market and the experimental session will end. There will be at least 20 periods in the experiment. There is a 1 in 5 chance that period 20 will be the last period. For every period after 20, the probability of a random stop is 1 in 5.

***Your Name and ID Number***

Before the practice trading period, the computer will ask for your name and ID number. This information is kept confidential, but it is important to the funding agency as proof of your participation. The bids and earnings of people in the experiment are confidential. Please do not look at someone else's screen and do not speak to another participant once the experiment begins. You may ask the experimenter questions at any time during the experiment. Are there any questions before we conduct the practice trading period?

## **Product Seizure treatment instructions**

***Introduction***

This is an experiment in the economics of market decision making. In this experiment, we will set up a market in which some of you will be BUYERS and some of you will be SELLERS.

The commodity you are trading is referred to as a "unit". Sellers make earnings by producing units at a cost and selling these units to buyers. Buyers make earnings by purchasing units from sellers and then redeeming (or reselling) these units to the experimenter. Earnings are recorded in a fictitious currency called tokens. Tokens are exchanged for cash at the rate of **100 tokens = $1.00**. Your earnings will be paid to you in CASH at the end of the experiment. To begin, every seller and buyer will be given an initial balance of **1500 tokens ($15.00)**. You may keep this money PLUS any you earn.

Buyers and sellers will be randomly paired and will exchange units for tokens in computerized markets over a sequence of trading periods. Each trading period consists of three one-minute bargaining rounds during which pairs of buyers and sellers negotiate trading prices.

All trading is conducted over the computer network. Each trading period will consist of a production period followed by random pairings of buyers and sellers and negotiations for trade prices. During the production period, the sellers determine the number of units to produce and the buyers wait. After the sellers have produced the units, buyers and sellers are randomly paired in each of the three one-minute bargaining rounds during which each buyer and seller pair negotiate for trade prices. See the figure below

BUYERS WAIT

BARGAINING CONVERT TOKEN

ROUNDS EARNINGS TO

DOLLARS AND CENTS

SELLERS PRODUCE UNITS

For the units produced, the cost of production is deducted from the seller's token balance. In addition, the computer will automatically account for sales or purchases that you have made and adjust your token balance accordingly. A listing of sales or purchases you have made and your adjusted token balance will be displayed on the computer screen at the end of every trading period. After you have viewed this information and clicked on OK, a new trading period with three trading rounds will begin. This experiment will consist of several trading periods. We will conduct a practice trading period to familiarize you with the mechanics of the computerized market before the actual experiment begins. During the practice trading period the information you see will be different than that in the actual experiment.

***Specific Instructions to Buyers***

During each trading period you are free to purchase up to 8 units. For the first unit that you buy during a trading period, you will receive the amount listed under UNIT VALUE for Unit 1. In this example, this amount is 55 tokens. For the second unit that you buy you will receive the amount listed under UNIT VALUE for Unit 2, which is 50 tokens. The redemption values for these and subsequent units will be displayed on your computer screen.

The earnings from each unit that you purchase (which are yours to keep) are computed by taking the difference between the redemption value and purchase price of the unit bought. That is,

**Your Earnings = Redemption Value - Purchase Price.**

Suppose, for example, that you buy 2 units in a trading period. If you pay 40 tokens for the first unit and 30 tokens for the second unit your earnings are:

**earnings for Unit 1 = 55 - 40 = 15**

**earnings for Unit 2 = 50 - 30 = 20.**

Your total earnings are:

**total earnings = 15 + 20 = 35 tokens.**

During the experiment this trading information will be summarized on the computer screen at the end of each trading period. Buyers also should be aware that they will not be allowed to spend more tokens buying units than what they have in their beginning balance in any one period.

***Specific Instructions to Sellers***

During each trading period you are free to sell up to 8 units. Remember, you must decide on the number of units you wish to produce and then sell in the production period. There is a 20% chance that, even after you successfully produced a specific unit and incurred the cost, the unit will “FAIL” and cannot be sold. **If you are a seller, you could see more than one of your units fail in some periods and no unit failures in other periods. However, know that overall, the chance that a unit fails is approximately 20%.** If a unit fails, you incur production costs but cannot sell the unit. Because you must decide on the number of units to produce before you sell them in the production period, you will also incur production costs for all units you produce, even the ones that fail.

The first unit that you produce during a trading period will cost you the amount listed under UNIT COST for Unit 1. In this example, this cost is 15 tokens. Unit 1's unit cost is 15 tokens. The second unit that you produce will cost you the amount listed under UNIT COST for Unit 2, which is 20 tokens and Unit 3 is 25 tokens. The unit costs for these and subsequent units will be displayed on your computer screens. Remember that there is a 20% chance that a unit will fail and cannot sold.

The earnings from each unit that you sell (which are yours to keep) are computed by taking the difference between the sale price and unit cost of the unit sold. That is,

**Your Earnings = Sale Price - Unit Cost**

Let's suppose that in the trading period there are no production failures. You sell Unit 1 for 45 tokens, Unit 2 for 40 tokens and Unit 3 for 35 tokens. Your earnings would then be:

**earnings for Unit 1 = 45 - 15 = 30**

**earnings for Unit 2 = 40 - 20 = 20**

**earnings for Unit 3 = 35 - 25 = 10**

Recall that there is a 20% chance that each unit fails. If no units fail, your total earnings are:

**total earnings = 30 + 20 + 10 = 60 tokens**

If Unit 3 fails you cannot sell it but you pay the cost. In this case your total earnings will be the 50 tokens that you receive from the first and second trades minus the 25 tokens you lose in production costs of Unit 3:

**total earnings = 30 + 20 - 25 = 25 tokens**

During the experiment this trading information will be summarized on the computer screen at the end of each trading period. If a produced unit has failed, it will be listed on the seller’s screens as “FAILED” when the trading period has ended. Sellers also should be aware that they will not be allowed to incur a production cost greater than the amount in their beginning token balance in any one period.

***Trading Rules for the Market***

Only one unit may be bought and sold at a time. A buyer makes bids to the seller to purchase a unit. A "bid" is a proposed price at which a buyer is willing to purchase a unit. Bids must become progressively higher. In other words, if the first bid for a unit is 50 tokens, then the second bid must be higher than 50. Suppose the second bid is 55 tokens, then the third bid must be higher than 55, and so on.

A seller makes offers to the buyer to sell a unit. An "offer" is a proposed price at which a seller is willing to sell a unit. Offers must become progressively lower. In other words, if the first offer to sell a unit is for 60 tokens, then the second offer must be lower than 60. Suppose the second offer is 55 tokens, then the third offer must be less than 55, and so on.

There is one further set of restrictions on bids and offers. The reason for these restrictions is just common sense. A buyer's bid cannot be higher than what is labeled on the computer screen as the BEST OFFER. In other words, a buyer cannot attempt to pay a price that is higher than that for which the seller is willing to sell. Similarly, a seller's offer cannot be lower than what is labeled as the BEST BID. In other words, a seller cannot attempt to sell at a price below that which the buyer is willing to pay. In fact, the computer will not allow such bids and offers.

After a trade has been made, bid and offer values are cleared from the screen. A buyer and seller pair may then resume entering bids and offers for additional units. Trades are made between buyer and seller pairs for one minute. After a minute has elapsed, buyers and sellers are again randomly paired and the next trading round begins.

Each trading period has a maximum time limit of 3 minutes or three one-minute trading rounds. A trading period will be terminated automatically if profitable trades cannot be made by the randomly matched buyer and seller. Because buyers incur a purchase price for units and sellers incur costs to produce units for sale, it is possible to reach a token balance of 0. If a player reaches a 0 token balance, they cannot continue to buy or produce units in the market and the experimental session will end. There will be at least 20 periods in the experiment. There is a 1 in 5 chance that period 20 will be the last period. For every period after 20, the probability of a random stop is 1 in 5.

***Your Name and ID Number***

Before the practice trading period, the computer will ask for your name and ID number. This information is kept confidential, but it is important to the funding agency as proof of your participation. The bids and earnings of people in the experiment are confidential. Please do not look at someone else’s screen and do not speak to another participant once the experiment begins. You may ask the experimenter questions at any time during the experiment. Are there any questions before we conduct the practice trading period?

## **Trade Seizure treatment instructions**

***Introduction***

This is an experiment in the economics of market decision making. In this experiment, we will set up a market in which some of you will be BUYERS and some of you will be SELLERS.

The commodity you are trading is referred to as a "unit". Sellers make earnings by producing units at a cost and selling these units to buyers. Buyers make earnings by purchasing units from sellers and then redeeming (or reselling) these units to the experimenter. Earnings are recorded in a fictitious currency called tokens. Tokens are exchanged for cash at the rate of **100 tokens = $1.00**. Your earnings will be paid to you in CASH at the end of the experiment. To begin, every seller and buyer will be given an initial balance of **1500 tokens ($15.00)**. You may keep this money PLUS any you earn.

Buyers and sellers will be randomly paired and will exchange units for tokens in computerized markets over a sequence of trading periods. Each trading period consists of three one-minute bargaining rounds during which pairs of buyers and sellers negotiate trading prices.

All trading is conducted over the computer network. Each trading period will consist of a production period followed by random pairings of buyers and sellers and negotiations for trade prices. During the production period, the sellers determine the number of units to produce and the buyers wait. After the sellers have produced the units, buyers and sellers are randomly paired in each of the three one-minute bargaining rounds during which each buyer and seller pair negotiate for trade prices. See the figure below

BUYERS WAIT

BARGAINING CONVERT TOKEN

ROUNDS EARNINGS TO

DOLLARS AND CENTS

SELLERS PRODUCE UNITS

For the units produced, the cost of production is deducted from the seller's token balance. In addition, the computer will automatically account for sales or purchases that you have made and adjust your token balance accordingly. A listing of sales or purchases you have made and your adjusted token balance will be displayed on the computer screen at the end of every trading period. After you have viewed this information and clicked on OK, a new trading period with three trading rounds will begin. This experiment will consist of several trading periods. We will conduct a practice trading period to familiarize you with the mechanics of the computerized market before the actual experiment begins. During the practice trading period the information you see will be different than that in the actual experiment.

***Specific Instructions to Buyers***

During each trading period you are free to purchase up to 8 units. **There is a 20% chance that, even after you successfully negotiate a trade price on a specific unit, the trade will not be completed. If a trade “FAILS”, you do not receive the unit, and you do not incur any purchase costs nor earn any tokens. Buyers could see more than one trade fail in some periods and no trade fail in other periods. However, know that overall, the chance that a trade fails is approximately 20%.** For the first unit that you buy during a trading period, you will receive the amount listed under UNIT VALUE for Unit 1. In this example, this amount is 55 tokens. For the second unit that you buy you will receive the amount listed under UNIT VALUE for Unit 2, which is 50 tokens. The redemption values for these and subsequent units will be displayed on your computer screen.

The earnings from each unit that you purchase (which are yours to keep) are computed by taking the difference between the redemption value and purchase price of the unit bought. That is,

**Your Earnings = Redemption Value - Purchase Price**

Suppose, for example, that you buy 2 units in a trading period. If you pay 40 tokens for the first unit and 30 tokens for the second unit your earnings are:

**earnings for Unit 1 = 55 - 40 = 15**

**earnings for Unit 2 = 50 - 30 = 20**

Recall that there is a 20% chance that each trade fails. If no trades fail, your total earnings are:

**total earnings = 15 + 20 = 35 tokens**

If the first trade fails, your total earnings will be only the 20 tokens that you receive from the first trade:

**total earnings = 0 + 20 = 20 tokens**

During the experiment this trading information will be summarized on the computer screen at the end of each trading period. If a trade has failed, it will be listed on the buyer’s screen as “FAILED” when the trading period has ended. Buyers also should be aware that they will not be allowed to spend more tokens buying units than what they have in their beginning balance in any one period.

***Specific Instructions to Sellers***

You must decide on the number of units you wish to produce and then sell in the production period. During each trading period you are free to sell up to 8 units. **There is a 20% chance that, even after you successfully negotiate a trade price on a specific unit, the trade will not be completed. If a trade “FAILS”, you do not sell the unit, nor earn any tokens. You still incur the cost of producing the unit and it is not available for sale in later bargaining rounds. Sellers could see more than one trade fail in some periods and no trade fail in other periods. However, know that overall, the chance that a trade fails is approximately 20%.** Remember if a trade is not completed, you incur production costs and do not sell the unit or receive the sale price.

Because you must decide on the number of units to produce before you sell them in the production period, you will also incur production costs for all units you produce, even the ones you do not sell in the trading period. The first unit that you sell during a trading period will cost you the amount listed under UNIT COST for Unit 1. In this example, this cost is 15 tokens. Unit 1's unit cost is 15 tokens. The second unit that you sell will cost you the amount listed under UNIT COST for Unit 2, which is 20 tokens and Unit 3 is 25 tokens. The unit costs for these and subsequent units will be displayed on your computer screens.

The earnings from each unit that you sell (which are yours to keep) are computed by taking the difference between the sale price and unit cost of the unit sold. That is,

**Your Earnings = Sale Price - Unit Cost**

Let's suppose that in the trading period you sell Unit 1 for 45 tokens, Unit 2 for 40 tokens and Unit 3 for 35 tokens. Your earnings would then be:

**earnings for Unit 1 = 45 - 15 = 30**

**earnings for Unit 2 = 40 - 20 = 20**

**earnings for Unit 3 = 35 - 25 = 10**

Recall that there is a 20% chance that each trade fails. If no trades fail, your total earnings are:

**total earnings = 30 + 20 + 10 = 60 tokens**

If the third trade fails, your total earnings will be the 50 tokens that you receive from the first and second trades minus the 25 tokens you lose in production costs of Unit 3:

**total earnings = 30 + 20 - 25 = 25 tokens**

During the experiment this trading information will be summarized on the computer screen at the end of each trading period. If a trade has failed, it will be listed on the seller’s screens as “FAILED” when the trading period has ended. Sellers also should be aware that they will not be allowed to incur a production cost greater than the amount in their beginning token balance in any one period.

***Trading Rules for the Market***

Only one unit may be bought and sold at a time. A buyer makes bids to the seller to purchase a unit. A "bid" is a proposed price at which a buyer is willing to purchase a unit. Bids must become progressively higher. In other words, if the first bid for a unit is 50 tokens, then the second bid must be higher than 50. Suppose the second bid is 55 tokens, then the third bid must be higher than 55, and so on.

A seller makes offers to the buyer to sell a unit. An "offer" is a proposed price at which a seller is willing to sell a unit. Offers must become progressively lower. In other words, if the first offer to sell a unit is for 60 tokens, then the second offer must be lower than 60. Suppose the second offer is 55 tokens, then the third offer must be less than 55, and so on.

There is one further set of restrictions on bids and offers. The reason for these restrictions is just common sense. A buyer's bid cannot be higher than what is labeled on the computer screen as the BEST OFFER. In other words, a buyer cannot attempt to pay a price that is higher than that for which the seller is willing to sell. Similarly, a seller's offer cannot be lower than what is labeled as the BEST BID. In other words, a seller cannot attempt to sell at a price below that which the buyer is willing to pay. In fact, the computer will not allow such bids and offers.

After a seller and buyer have made a trade, the trading price will be listed on both the buyer's and seller's screens. If a trade has failed, it will be listed on both the buyer’s and seller’s screens as “FAILED” when the trading period has ended. After a trade has been made, bid and offer values are cleared from the screen. A buyer and seller pair may then resume entering bids and offers for additional units. Trades are made between buyer and seller pairs for one minute. After a minute has elapsed, buyers and sellers are again randomly paired and the next trading round begins.

Each trading period has a maximum time limit of 3 minutes or three one-minute trading rounds. A trading period will be terminated automatically if profitable trades cannot be made by the randomly matched buyer and seller. Because buyers incur a purchase price for units and sellers incur costs to produce units for sale, it is possible to reach a token balance of 0. If a player reaches a 0 token balance, they cannot continue to buy or produce units in the market and the experimental session will end. There will be at least 20 periods in the experiment. There is a 1 in 5 chance that period 20 will be the last period. For every period after 20, the probability of a random stop is 1 in 5.

***Your Name and ID Number***

Before the practice trading period, the computer will ask for your name and ID number. This information is kept confidential, but it is important to the funding agency as proof of your participation. The bids and earnings of people in the experiment are confidential. Please do not look at someone else’s screen and do not speak to another participant once the experiment begins. You may ask the experimenter questions at any time during the experiment. Are there any questions before we conduct the practice trading period?
