# Supplementary material for: The relative effectiveness of law enforcement policies aimed at reducing illegal trade: Evidence from laboratory markets
Source: PLoS One. 2021 Nov 2;16(11):e0259254. doi: 10.1371/journal.pone.0259254 (PMC8562810; doi:10.1371/journal.pone.0259254)
Supplement: S2 Text — (DOCX) [file pone.0259254.s002.docx]

# **Supporting information: S2 Text. Wilcoxon’s non-parametric Rank-Sum and regression results**

#

## **Wilcoxon’s non-parametric Rank-Sum Test**

To lend validity to the convergence analysis results, treatment effects were also tested using Wilcoxon’s non-parametric Rank-Sum tests for all variables (Units Produced, Units Traded, Price, Seller Earnings, Buyer Earnings, and Total Earnings). Since autocorrelation can occur when pooling data across all periods in the experiment, we perform the Wilcoxon’s non-parametric Rank-Sum test on the last five periods, as is common in the literature [30-31,50]. The assumption of using only the last five periods for analysis is that any learning that occurred in early periods is eliminated and behavior becomes stable. Results from the Wilcoxon’s non-parametric Rank-Sum tests are consistent with those generated by the convergence analysis (Table 4; Table A). The values in Table A for the variable, Units Produced are those reported in Table 4. The only differences between the Wilcoxon’s non-parametric Rank-Sum test results and those from the convergence analysis are with regard to the variable, Price. The Wilcoxon’s non-parametric Rank-Sum test did find a significant difference between the Seller Profit Seizure and No Seizure treatments, whereas the convergence analysis found none. Further the Wilcoxon’s non-parametric Rank-Sum test did not find a significant treatment effect for the Buyer Seizure and Both Seizure treatments, whereas a significant difference was found from the convergence analysis. Lastly, the mean Price in the Product Seizure treatment is less than that in the No Seizure treatment when focusing on only the last five periods, but is significantly more than the No Seizure treatment in the convergence analysis. These differences do not change the main results and provide validity to the main conclusions drawn from the convergence analysis.

**Table A. Wilcoxon’s Non-Parametric Rank-Sum Tests Measuring Treatment Differences from the No Seizure Treatment**

|  |  |  |  |  |  |  |  |  |  |
| --- | --- | --- | --- | --- | --- | --- | --- | --- | --- |
|  | **Units Produced** | **Units Traded** | | **Price** | **Seller Earnings** | **Buyer Earnings Total Earnings** | | | |
| **Difference from the No Seizure Treatment** | **Mean Difference**  **(P-value)** | **Mean Difference**  **(P-value)** | **Mean Difference**  **(P-value)** | | **Mean Difference**  **(P-value)** | | **Mean Difference**  **(P-value)** | **Mean Difference**  **(P-value)** |  |
| **Seller Profit Seizure** | -0.16  (0.367) | 0.21  (0.911) | -5.36*  (0.016) | | -8.51*  (0.005) | | 2.55  (0.092) | -23.86*  (0.011) |  |
| **Buyer Profit Seizure** | -1.33  (0.099) | -0.18*  (0.014) | -5.32  (0.084) | | -5.65  (0.071) | | -8.42*  (0.001) | -56.28*  (0.001) |  |
| **Both Profit Seizure** | 0.94  (0.197) | 0.50*  (0.011) | -3.86  (0.378) | | -3.65  (0.067) | | -10.55*  (0.001) | -56.81*  (0.001) |  |
| **Product Seizure** | 0.57  (0.409) | -0.52*  (0.001) | -2.63*  (0.014) | | -11.26*  (0.001) | | -9.66*  (0.001) | -83.89*  (0.001) |  |
| **Trade Seizure** | -3.30*  (0.022) | -0.40*  (0.001) | -4.88  (0.882) | | -20.09*  (0.001) | | -13.99*  (0.001) | -136.32*  (0.001) |  |

* Indicates a significant difference between the given treatment and the No Seizure treatment at the 5% significance level.

^i^ Difference between the treatment of interest and the No Seizure treatment are reported. A positive value shows that the mean value of the treatment of interest is larger than the mean of the No Seizure treatment for the variable listed. A negative value indicates that the No Seizure treatment mean value is larger than the treatment of interest for the variable listed.

## **Regression Analysis**

To further provide validity to the convergence analysis results, regression analyses were conducted using data from the last five periods of the market experiment. Including covariates when testing for treatment effect leads to biased estimators [51], but properly adjusting for any imbalanced prognostic variables is appropriate [52-53]. Previous research has found that gender influences individual behavior in similar market experiments [41]. If gender is found to be imbalanced across treatments, it is appropriate to include this as a covariate in regression analyses.

Every attempt was made to fully randomize participants across the treatments. To determine if the randomization of participants across treatments was sufficiently successful, we follow the method outlined by Imbens and Rubin [51] and calculate normalized differences in means (NDM) for gender. Since the data used in all analyses is at the session level, the proportion of women in a session was used to test for imbalance.

We calculated the NDM for each treatment compared to the No Seizure treatment. The NDM for the proportion of women between the No Seizure treatment and any other treatment is as follows:

$NDM=\frac{X_{1}-X_{i}}{\sqrt{\frac{s_{1}^{2}+s_{i}^{2}}{2}}}$ (1)

where $X_{1}$ is the No Seizure treatment’s sample mean and $s_{1}^{2}$ is the No Seizure treatment’s variance for the proportion of women per session. $X_{i}$ is treatment *i*’s sample mean and $s_{i}^{2}$ is treatment *i*’s variance for the proportion of women per session (*i*=Seller Profit, Buyer Profit, Both Profit, Product, Trade). As outlined by Imbens and Rubin [51], when NDM>|0.25|, the proportion of women per session should be considered in the analysis of treatment effects. Based on equation 1, we find that the proportion of women per session can be included as a covariate in the regression analysis (Table B).

**Table B. Percent of Women in a Session Balance Analysis Across Treatments**

|  | **Balance Test for Percent of Women in a Session** | | | |
| --- | --- | --- | --- | --- |
| **Treatment** | **SD** | **Variance** | **Mean** | **NDM** |
|  |  |  |  |  |
| No Seizure | 0.162 | 0.026 | 0.500 | - |
| Seller Profit Seizure | 0.062 | 0.004 | 0.426 | 0.603 |
| Buyer Profit Seizure | 0.119 | 0.014 | 0.333 | 1.175 |
| Both Profit Seizure | 0.061 | 0.004 | 0.349 | 1.234 |
| Product Seizure | 0.173 | 0.030 | 0.427 | 0.436 |
| Trade Seizure | 0.197 | 0.039 | 0.521 | -0.116 |
|  |  |  |  |  |

Regression analyses performed on the last five periods support the results from the convergence analysis (Table 4, Table C). The only notable difference between the regression analyses and the convergence analysis is that the regression analysis found the Both Profit Seizure treatment had significantly more Units Produced than the No Seizure treatment, where this was not found to be significant in the convergence analysis.

**Table C. Regression Analysis on Last Five Periods Across Treatments**

|  | **Dependent Variable** | | | | | |
| --- | --- | --- | --- | --- | --- | --- |
| **Independent Variable**  **(No Seizure is the Omitted treatment)** | **Units Produced** | **Units Traded** | **Price** | **Seller Earnings** | **Buyer Earnings** | **Total Earnings** |
| Seller Profit Seizure | 0.096 | 0.598 | -1.631 | -15.410* | 5.072 | -41.7 |
| Buyer Profit Seizure | -0.476 | -0.444 | -1.532 | -6.902 | -23.82* | -123.2* |
| Both Profit Seizure | 1.299* | 1.719* | 3.502* | -1.597 | -28.60* | -121.1* |
| Product Seizure | 0.815 | -2.461* | 4.692* | -43.170* | -28.78* | -288.1* |
| Trade Seizure | -3.203* | -2.648* | -1.205 | -52.240* | -46.08* | -393.6* |
| Percent Women (in Session) | 3.729* | 3.752* | 11.89* | 40.34* | -8.39 | 127.7* |
| Constant | 13.46* | 12.75* | 69.37* | 82.35* | 143.40* | 903.40* |
| F-stat | 16.81 | 18.18 | 6.15 | 14.67 |  |  |
| N | 216 | 216 | 216 | 216 | 216 | 216 |

******* Indicates significance at the 5% level
