# Supplementary material for: The relative effectiveness of law enforcement policies aimed at reducing illegal trade: Evidence from laboratory markets
Source: PLoS One. 2021 Nov 2;16(11):e0259254. doi: 10.1371/journal.pone.0259254 (PMC8562810; doi:10.1371/journal.pone.0259254)
Supplement: S3 Text — (DOCX) [file pone.0259254.s003.docx]

# **Supporting Information: S3 Text. Data and SAS and STATA code**

## **Data**

The data reported in Table D is the average over the eight participants in a given session of each variable. This data is used in both the SAS convergence analysis and the STATA Wilcoxon Rank-Sum tests and regression analyses. A Treatment value of 0 indicates the No Seizure treatment, a value of 1 indicates the Product Seizure treatment, a value of 2 indicates the Trade Seizure treatment, a value of 3 indicates the Seller Profit Seizure treatment, a value of 4 indicates the Buyer Seizure treatment, and a value of 5 indicates the Both Seizure treatment.

**Table D. Study Data Averages per Session**

| Period | Session | Treatment | Units Produced | Units Traded | Price | Seller earning | Buyer earnings | Total Earnings | Proportion Women |
| --- | --- | --- | --- | --- | --- | --- | --- | --- | --- |
| 1 | 1 | 3 | 20 | 20 | 37 | -88 | 345 | 1028 | 0.5 |
| 2 | 1 | 3 | 25 | 21 | 42 | -149 | 329 | 720 | 0.5 |
| 3 | 1 | 3 | 21 | 18 | 63 | -10 | 196 | 744 | 0.5 |
| 4 | 1 | 3 | 23 | 20 | 67 | 4 | 212 | 864 | 0.5 |
| 5 | 1 | 3 | 22 | 19 | 63 | -13 | 210 | 788 | 0.5 |
| 6 | 1 | 3 | 15 | 15 | 68 | 58 | 157 | 860 | 0.5 |
| 7 | 1 | 3 | 14 | 14 | 66 | 57 | 167 | 896 | 0.5 |
| 8 | 1 | 3 | 13 | 13 | 66 | 58 | 163 | 884 | 0.5 |
| 9 | 1 | 3 | 15 | 15 | 61 | 54 | 198 | 1008 | 0.5 |
| 10 | 1 | 3 | 15 | 15 | 64 | 59 | 182 | 964 | 0.5 |
| 11 | 1 | 3 | 18 | 17 | 65 | 49 | 176 | 900 | 0.5 |
| 12 | 1 | 3 | 17 | 16 | 65 | 51 | 173 | 896 | 0.5 |
| 13 | 1 | 3 | 16 | 16 | 68 | 75 | 161 | 944 | 0.5 |
| 14 | 1 | 3 | 15 | 15 | 64 | 60 | 186 | 984 | 0.5 |
| 15 | 1 | 3 | 16 | 16 | 66 | 63 | 186 | 996 | 0.5 |
| 16 | 1 | 3 | 15 | 15 | 68 | 69 | 172 | 964 | 0.5 |
| 17 | 1 | 3 | 17 | 17 | 68 | 71 | 172 | 972 | 0.5 |
| 18 | 1 | 3 | 15 | 15 | 65 | 65 | 189 | 1016 | 0.5 |
| 19 | 1 | 3 | 18 | 18 | 67 | 68 | 175 | 972 | 0.5 |
| 20 | 1 | 3 | 14 | 14 | 68 | 71 | 146 | 868 | 0.5 |
| 1 | 2 | 3 | 12 | 12 | 71 | 77 | 132 | 836 | 0.375 |
| 2 | 2 | 3 | 15 | 12 | 71 | 37 | 142 | 716 | 0.375 |
| 3 | 2 | 3 | 13 | 11 | 79 | 68 | 107 | 700 | 0.375 |
| 4 | 2 | 3 | 12 | 12 | 79 | 95 | 99 | 776 | 0.375 |
| 5 | 2 | 3 | 13 | 12 | 74 | 73 | 131 | 816 | 0.375 |
| 6 | 2 | 3 | 14 | 14 | 77 | 98 | 136 | 936 | 0.375 |
| 7 | 2 | 3 | 15 | 15 | 78 | 104 | 125 | 916 | 0.375 |
| 8 | 2 | 3 | 14 | 14 | 79 | 102 | 123 | 900 | 0.375 |
| 9 | 2 | 3 | 16 | 13 | 80 | 58 | 93 | 604 | 0.375 |
| 10 | 2 | 3 | 15 | 13 | 76 | 66 | 118 | 736 | 0.375 |
| 11 | 2 | 3 | 14 | 12 | 75 | 60 | 130 | 760 | 0.375 |
| 12 | 2 | 3 | 13 | 13 | 81 | 102 | 101 | 812 | 0.375 |
| 13 | 2 | 3 | 14 | 14 | 76 | 96 | 132 | 912 | 0.375 |
| 14 | 2 | 3 | 14 | 14 | 72 | 83 | 152 | 940 | 0.375 |
| 15 | 2 | 3 | 14 | 13 | 78 | 85 | 116 | 804 | 0.375 |
| 16 | 2 | 3 | 13 | 13 | 74 | 82 | 136 | 872 | 0.375 |
| 17 | 2 | 3 | 14 | 14 | 75 | 90 | 141 | 924 | 0.375 |
| 18 | 2 | 3 | 14 | 14 | 75 | 95 | 147 | 968 | 0.375 |
| 19 | 2 | 3 | 14 | 13 | 77 | 81 | 131 | 848 | 0.375 |
| 20 | 2 | 3 | 14 | 14 | 76 | 97 | 136 | 932 | 0.375 |
| 1 | 3 | 3 | 17 | 17 | 85 | 133 | 118 | 1004 | 0.375 |
| 2 | 3 | 3 | 21 | 20 | 83 | 115 | 118 | 932 | 0.375 |
| 3 | 3 | 3 | 20 | 20 | 84 | 136 | 116 | 1008 | 0.375 |
| 4 | 3 | 3 | 19 | 19 | 89 | 153 | 98 | 1004 | 0.375 |
| 5 | 3 | 3 | 21 | 21 | 86 | 145 | 113 | 1032 | 0.375 |
| 6 | 3 | 3 | 19 | 19 | 92 | 166 | 86 | 1008 | 0.375 |
| 7 | 3 | 3 | 19 | 18 | 92 | 149 | 80 | 916 | 0.375 |
| 8 | 3 | 3 | 19 | 19 | 90 | 154 | 96 | 1000 | 0.375 |
| 9 | 3 | 3 | 19 | 19 | 94 | 176 | 75 | 1004 | 0.375 |
| 10 | 3 | 3 | 19 | 19 | 91 | 156 | 84 | 960 | 0.375 |
| 11 | 3 | 3 | 19 | 19 | 93 | 168 | 79 | 988 | 0.375 |
| 12 | 3 | 3 | 19 | 19 | 91 | 157 | 90 | 988 | 0.375 |
| 13 | 3 | 3 | 19 | 18 | 92 | 144 | 88 | 928 | 0.375 |
| 14 | 3 | 3 | 19 | 19 | 92 | 166 | 74 | 960 | 0.375 |
| 15 | 3 | 3 | 19 | 19 | 91 | 162 | 82 | 976 | 0.375 |
| 16 | 3 | 3 | 19 | 19 | 87 | 147 | 109 | 1024 | 0.375 |
| 17 | 3 | 3 | 19 | 19 | 88 | 151 | 81 | 928 | 0.375 |
| 18 | 3 | 3 | 19 | 19 | 89 | 156 | 91 | 988 | 0.375 |
| 19 | 3 | 3 | 19 | 18 | 91 | 142 | 90 | 928 | 0.375 |
| 20 | 3 | 3 | 22 | 22 | 86 | 149 | 107 | 1024 | 0.375 |
| 1 | 4 | 3 | 15 | 15 | 62 | 49 | 194 | 972 | 0.5 |
| 2 | 4 | 3 | 17 | 16 | 62 | 39 | 203 | 968 | 0.5 |
| 3 | 4 | 3 | 16 | 14 | 54 | -2 | 213 | 844 | 0.5 |
| 4 | 4 | 3 | 17 | 14 | 59 | -2 | 196 | 776 | 0.5 |
| 5 | 4 | 3 | 16 | 16 | 63 | 59 | 202 | 1044 | 0.5 |
| 6 | 4 | 3 | 15 | 12 | 62 | 3 | 167 | 680 | 0.5 |
| 7 | 4 | 3 | 15 | 13 | 61 | 29 | 181 | 840 | 0.5 |
| 8 | 4 | 3 | 13 | 13 | 61 | 51 | 178 | 916 | 0.5 |
| 9 | 4 | 3 | 15 | 15 | 65 | 61 | 181 | 968 | 0.5 |
| 10 | 4 | 3 | 14 | 14 | 64 | 61 | 160 | 884 | 0.5 |
| 11 | 4 | 3 | 11 | 11 | 65 | 58 | 146 | 816 | 0.5 |
| 12 | 4 | 3 | 11 | 11 | 70 | 65 | 136 | 804 | 0.5 |
| 13 | 4 | 3 | 11 | 11 | 72 | 71 | 129 | 800 | 0.5 |
| 14 | 4 | 3 | 13 | 13 | 63 | 57 | 184 | 964 | 0.5 |
| 15 | 4 | 3 | 12 | 12 | 74 | 80 | 128 | 832 | 0.5 |
| 16 | 4 | 3 | 12 | 12 | 71 | 70 | 143 | 852 | 0.5 |
| 17 | 4 | 3 | 12 | 12 | 71 | 77 | 133 | 840 | 0.5 |
| 18 | 4 | 3 | 12 | 12 | 74 | 80 | 135 | 860 | 0.5 |
| 19 | 4 | 3 | 12 | 12 | 76 | 86 | 125 | 844 | 0.5 |
| 20 | 4 | 3 | 12 | 12 | 75 | 82 | 127 | 836 | 0.5 |
| 1 | 5 | 3 | 16 | 15 | 48 | -6 | 247 | 964 | 0.43 |
| 2 | 5 | 3 | 18 | 15 | 53 | -41 | 230 | 756 | 0.43 |
| 3 | 5 | 3 | 16 | 16 | 56 | 30 | 220 | 1000 | 0.43 |
| 4 | 5 | 3 | 14 | 13 | 56 | 27 | 176 | 812 | 0.43 |
| 5 | 5 | 3 | 14 | 14 | 54 | 29 | 216 | 980 | 0.43 |
| 6 | 5 | 3 | 13 | 12 | 62 | 37 | 164 | 804 | 0.43 |
| 7 | 5 | 3 | 14 | 13 | 60 | 35 | 173 | 832 | 0.43 |
| 8 | 5 | 3 | 14 | 13 | 62 | 45 | 176 | 884 | 0.43 |
| 9 | 5 | 3 | 13 | 12 | 61 | 39 | 172 | 844 | 0.43 |
| 10 | 5 | 3 | 12 | 12 | 66 | 63 | 151 | 856 | 0.43 |
| 11 | 5 | 3 | 13 | 13 | 62 | 53 | 167 | 880 | 0.43 |
| 12 | 5 | 3 | 13 | 13 | 67 | 68 | 166 | 936 | 0.43 |
| 13 | 5 | 3 | 12 | 12 | 66 | 63 | 153 | 864 | 0.43 |
| 14 | 5 | 3 | 13 | 12 | 66 | 48 | 148 | 784 | 0.43 |
| 15 | 5 | 3 | 13 | 12 | 68 | 56 | 137 | 772 | 0.43 |
| 16 | 5 | 3 | 13 | 13 | 64 | 61 | 169 | 920 | 0.43 |
| 17 | 5 | 3 | 13 | 13 | 61 | 51 | 179 | 920 | 0.43 |
| 18 | 5 | 3 | 13 | 12 | 62 | 41 | 159 | 800 | 0.43 |
| 19 | 5 | 3 | 13 | 13 | 68 | 70 | 161 | 924 | 0.43 |
| 20 | 5 | 3 | 12 | 12 | 64 | 58 | 149 | 828 | 0.43 |
| 1 | 6 | 3 | 15 | 14 | 71 | 68 | 155 | 892 | 0.375 |
| 2 | 6 | 3 | 21 | 18 | 66 | 2 | 182 | 736 | 0.375 |
| 3 | 6 | 3 | 18 | 18 | 67 | 67 | 179 | 984 | 0.375 |
| 4 | 6 | 3 | 18 | 18 | 69 | 67 | 181 | 992 | 0.375 |
| 5 | 6 | 3 | 17 | 16 | 68 | 57 | 182 | 956 | 0.375 |
| 6 | 6 | 3 | 17 | 17 | 69 | 73 | 171 | 976 | 0.375 |
| 7 | 6 | 3 | 16 | 16 | 71 | 81 | 152 | 932 | 0.375 |
| 8 | 6 | 3 | 19 | 19 | 66 | 58 | 187 | 980 | 0.375 |
| 9 | 6 | 3 | 17 | 17 | 66 | 62 | 190 | 1008 | 0.375 |
| 10 | 6 | 3 | 16 | 16 | 68 | 70 | 182 | 1008 | 0.375 |
| 11 | 6 | 3 | 17 | 16 | 72 | 73 | 150 | 892 | 0.375 |
| 12 | 6 | 3 | 17 | 17 | 70 | 74 | 173 | 988 | 0.375 |
| 13 | 6 | 3 | 16 | 16 | 69 | 75 | 158 | 932 | 0.375 |
| 14 | 6 | 3 | 18 | 18 | 68 | 72 | 196 | 1072 | 0.375 |
| 15 | 6 | 3 | 16 | 16 | 71 | 81 | 163 | 976 | 0.375 |
| 16 | 6 | 3 | 18 | 18 | 67 | 62 | 187 | 996 | 0.375 |
| 17 | 6 | 3 | 16 | 15 | 66 | 52 | 184 | 944 | 0.375 |
| 18 | 6 | 3 | 18 | 18 | 63 | 49 | 173 | 888 | 0.375 |
| 19 | 6 | 3 | 16 | 16 | 69 | 71 | 180 | 1004 | 0.375 |
| 20 | 6 | 3 | 17 | 16 | 68 | 57 | 178 | 940 | 0.375 |
| 1 | 1 | 0 | 16 | 14 | 66.43 | 45 | 177.5 | 890 | 0.25 |
| 2 | 1 | 0 | 15 | 15 | 70.73 | 97.75 | 167.25 | 1060 | 0.25 |
| 3 | 1 | 0 | 13 | 12 | 65.25 | 38.25 | 154.25 | 770 | 0.25 |
| 4 | 1 | 0 | 10 | 10 | 68.9 | 67.25 | 122.75 | 760 | 0.25 |
| 5 | 1 | 0 | 14 | 14 | 69.29 | 90 | 165 | 1020 | 0.25 |
| 6 | 1 | 0 | 15 | 15 | 73.07 | 99 | 143.5 | 970 | 0.25 |
| 7 | 1 | 0 | 19 | 18 | 65.89 | 46.5 | 188.5 | 940 | 0.25 |
| 8 | 1 | 0 | 15 | 14 | 66.36 | 59.75 | 155.25 | 860 | 0.25 |
| 9 | 1 | 0 | 13 | 13 | 72 | 99 | 151 | 1000 | 0.25 |
| 10 | 1 | 0 | 13 | 13 | 67.15 | 83.25 | 156.75 | 960 | 0.25 |
| 11 | 1 | 0 | 12 | 12 | 74.33 | 100.5 | 134.5 | 940 | 0.25 |
| 12 | 1 | 0 | 12 | 12 | 74.92 | 102.25 | 130.25 | 930 | 0.25 |
| 13 | 1 | 0 | 14 | 14 | 73 | 105.5 | 152 | 1030 | 0.25 |
| 14 | 1 | 0 | 14 | 14 | 75.79 | 115.25 | 144.75 | 1040 | 0.25 |
| 15 | 1 | 0 | 13 | 13 | 75.69 | 111 | 139 | 1000 | 0.25 |
| 16 | 1 | 0 | 13 | 13 | 78 | 118.5 | 131.5 | 1000 | 0.25 |
| 17 | 1 | 0 | 12 | 10 | 82.1 | 82.75 | 99.75 | 730 | 0.25 |
| 18 | 1 | 0 | 13 | 13 | 77 | 115.25 | 127.25 | 970 | 0.25 |
| 19 | 1 | 0 | 13 | 12 | 80.92 | 107.75 | 114.75 | 890 | 0.25 |
| 20 | 1 | 0 | 13 | 13 | 79.38 | 123 | 127 | 1000 | 0.25 |
| 1 | 2 | 0 | 16 | 16 | 68.06 | 89.75 | 185.25 | 1100 | 0.75 |
| 2 | 2 | 0 | 18 | 16 | 68.62 | 59.5 | 183 | 970 | 0.75 |
| 3 | 2 | 0 | 19 | 17 | 66.29 | 49.25 | 200.75 | 1000 | 0.75 |
| 4 | 2 | 0 | 17 | 17 | 66 | 83 | 199.5 | 1130 | 0.75 |
| 5 | 2 | 0 | 18 | 17 | 66.65 | 68.25 | 194.25 | 1050 | 0.75 |
| 6 | 2 | 0 | 17 | 17 | 67.59 | 89.75 | 190.25 | 1120 | 0.75 |
| 7 | 2 | 0 | 18 | 18 | 67.94 | 90.75 | 189.25 | 1120 | 0.75 |
| 8 | 2 | 0 | 17 | 17 | 68.06 | 91.75 | 190.75 | 1130 | 0.75 |
| 9 | 2 | 0 | 16 | 16 | 63.69 | 72.25 | 202.75 | 1100 | 0.75 |
| 10 | 2 | 0 | 15 | 15 | 66.8 | 85.5 | 182 | 1070 | 0.75 |
| 11 | 2 | 0 | 15 | 14 | 61.57 | 50.5 | 192 | 970 | 0.75 |
| 12 | 2 | 0 | 16 | 16 | 64.81 | 79.25 | 198.25 | 1110 | 0.75 |
| 13 | 2 | 0 | 16 | 16 | 64.88 | 79.5 | 198 | 1110 | 0.75 |
| 14 | 2 | 0 | 16 | 16 | 65.88 | 83.5 | 166.5 | 1000 | 0.75 |
| 15 | 2 | 0 | 16 | 16 | 64.19 | 76.75 | 185.75 | 1050 | 0.75 |
| 16 | 2 | 0 | 16 | 16 | 67.94 | 91.75 | 183.25 | 1100 | 0.75 |
| 17 | 2 | 0 | 16 | 15 | 66.27 | 68.5 | 181.5 | 1000 | 0.75 |
| 18 | 2 | 0 | 16 | 16 | 65.12 | 80.5 | 177 | 1030 | 0.75 |
| 19 | 2 | 0 | 16 | 14 | 64.07 | 44.25 | 175.75 | 880 | 0.75 |
| 20 | 2 | 0 | 16 | 15 | 67.67 | 73.75 | 176.25 | 1000 | 0.75 |
| 1 | 3 | 0 | 16 | 15 | 76.93 | 108.5 | 177.5 | 1144 | 0.625 |
| 2 | 3 | 0 | 15 | 14 | 76.64 | 100.75 | 167.25 | 1072 | 0.625 |
| 3 | 3 | 0 | 15 | 15 | 78.6 | 129.75 | 154.25 | 1136 | 0.625 |
| 4 | 3 | 0 | 15 | 14 | 71.93 | 86.75 | 122.75 | 838 | 0.625 |
| 5 | 3 | 0 | 14 | 13 | 72.85 | 86.75 | 165 | 1007 | 0.625 |
| 6 | 3 | 0 | 13 | 13 | 75.08 | 106.5 | 143.5 | 1000 | 0.625 |
| 7 | 3 | 0 | 14 | 14 | 77.57 | 121.5 | 188.5 | 1240 | 0.625 |
| 8 | 3 | 0 | 14 | 12 | 78.42 | 85.25 | 155.25 | 962 | 0.625 |
| 9 | 3 | 0 | 13 | 13 | 81.23 | 126.5 | 151 | 1110 | 0.625 |
| 10 | 3 | 0 | 15 | 15 | 83.33 | 145 | 156.75 | 1207 | 0.625 |
| 11 | 3 | 0 | 14 | 14 | 80.29 | 118.5 | 134.5 | 1012 | 0.625 |
| 12 | 3 | 0 | 16 | 16 | 81.38 | 143 | 130.25 | 1093 | 0.625 |
| 13 | 3 | 0 | 15 | 15 | 78.67 | 130 | 152 | 1128 | 0.625 |
| 14 | 3 | 0 | 16 | 16 | 78.06 | 129.75 | 144.75 | 1098 | 0.625 |
| 15 | 3 | 0 | 15 | 15 | 79.6 | 131 | 139 | 1080 | 0.625 |
| 16 | 3 | 0 | 18 | 18 | 77.33 | 128 | 131.5 | 1038 | 0.625 |
| 17 | 3 | 0 | 17 | 16 | 78.38 | 111 | 99.75 | 843 | 0.625 |
| 18 | 3 | 0 | 17 | 16 | 77.88 | 114 | 127.25 | 965 | 0.625 |
| 19 | 3 | 0 | 17 | 17 | 76 | 125.5 | 114.75 | 961 | 0.625 |
| 20 | 3 | 0 | 17 | 16 | 74.94 | 97.25 | 127 | 897 | 0.625 |
| 1 | 4 | 0 | 15 | 15 | 72 | 94 | 137 | 928 | 0.375 |
| 2 | 4 | 0 | 16 | 13 | 80 | 71 | 96 | 664 | 0.375 |
| 3 | 4 | 0 | 20 | 16 | 79 | 34 | 141 | 696 | 0.375 |
| 4 | 4 | 0 | 16 | 14 | 80 | 86 | 116 | 808 | 0.375 |
| 5 | 4 | 0 | 15 | 14 | 80 | 105 | 104 | 840 | 0.375 |
| 6 | 4 | 0 | 18 | 17 | 75 | 97 | 157 | 1016 | 0.375 |
| 7 | 4 | 0 | 20 | 15 | 79 | 34 | 127 | 648 | 0.375 |
| 8 | 4 | 0 | 18 | 13 | 86 | 55 | 94 | 600 | 0.375 |
| 9 | 4 | 0 | 20 | 17 | 78 | 64 | 148 | 848 | 0.375 |
| 10 | 4 | 0 | 19 | 17 | 79 | 98 | 139 | 944 | 0.375 |
| 11 | 4 | 0 | 17 | 15 | 83 | 106 | 116 | 888 | 0.375 |
| 12 | 4 | 0 | 18 | 17 | 71 | 78 | 176 | 1016 | 0.375 |
| 13 | 4 | 0 | 17 | 14 | 71 | 45 | 157 | 808 | 0.375 |
| 14 | 4 | 0 | 17 | 12 | 78 | 28 | 117 | 576 | 0.375 |
| 15 | 4 | 0 | 17 | 15 | 69 | 55 | 162 | 864 | 0.375 |
| 16 | 4 | 0 | 16 | 13 | 72 | 49 | 130 | 720 | 0.375 |
| 17 | 4 | 0 | 16 | 13 | 77 | 63 | 126 | 760 | 0.375 |
| 18 | 4 | 0 | 16 | 15 | 71 | 80 | 159 | 960 | 0.375 |
| 19 | 4 | 0 | 15 | 15 | 73 | 105 | 160 | 1056 | 0.375 |
| 20 | 4 | 0 | 15 | 15 | 74 | 108 | 144 | 1008 | 0.375 |
| 1 | 5 | 0 | 13 | 12 | 73 | 83 | 136 | 880 | 0.5 |
| 2 | 5 | 0 | 16 | 15 | 80 | 117 | 132 | 1000 | 0.5 |
| 3 | 5 | 0 | 15 | 14 | 76 | 93 | 142 | 936 | 0.5 |
| 4 | 5 | 0 | 15 | 14 | 75 | 95 | 145 | 960 | 0.5 |
| 5 | 5 | 0 | 16 | 14 | 76 | 78 | 139 | 864 | 0.5 |
| 6 | 5 | 0 | 15 | 14 | 78 | 90 | 124 | 856 | 0.5 |
| 7 | 5 | 0 | 14 | 13 | 75 | 83 | 131 | 856 | 0.5 |
| 8 | 5 | 0 | 16 | 15 | 82 | 125 | 124 | 1000 | 0.5 |
| 9 | 5 | 0 | 15 | 14 | 83 | 128 | 113 | 968 | 0.5 |
| 10 | 5 | 0 | 16 | 15 | 81 | 118 | 128 | 984 | 0.5 |
| 11 | 5 | 0 | 17 | 16 | 76 | 106 | 148 | 1016 | 0.5 |
| 12 | 5 | 0 | 14 | 14 | 77 | 121 | 138 | 1040 | 0.5 |
| 13 | 5 | 0 | 14 | 14 | 74 | 111 | 136 | 984 | 0.5 |
| 14 | 5 | 0 | 14 | 13 | 79 | 103 | 119 | 888 | 0.5 |
| 15 | 5 | 0 | 14 | 14 | 81 | 133 | 121 | 1016 | 0.5 |
| 16 | 5 | 0 | 14 | 14 | 79 | 126 | 128 | 1016 | 0.5 |
| 17 | 5 | 0 | 14 | 14 | 81 | 130 | 119 | 1000 | 0.5 |
| 18 | 5 | 0 | 14 | 13 | 79 | 108 | 126 | 936 | 0.5 |
| 19 | 5 | 0 | 14 | 14 | 76 | 117 | 142 | 1040 | 0.5 |
| 20 | 5 | 0 | 14 | 14 | 75 | 112 | 142 | 1016 | 0.5 |
| 1 | 6 | 0 | 16 | 15 | 72 | 87 | 158 | 980 | 0.5 |
| 2 | 6 | 0 | 17 | 15.5 | 70 | 74 | 162 | 944 | 0.5 |
| 3 | 6 | 0 | 15.5 | 14 | 76 | 92 | 130 | 888 | 0.5 |
| 4 | 6 | 0 | 17 | 15.5 | 71 | 76 | 160 | 944 | 0.5 |
| 5 | 6 | 0 | 15 | 13 | 70 | 62 | 147 | 836 | 0.5 |
| 6 | 6 | 0 | 16.5 | 15.5 | 72 | 88 | 154 | 968 | 0.5 |
| 7 | 6 | 0 | 15.5 | 15.5 | 77 | 126 | 130 | 1024 | 0.5 |
| 8 | 6 | 0 | 15 | 13.5 | 77 | 95 | 127 | 888 | 0.5 |
| 9 | 6 | 0 | 15.5 | 14.5 | 80 | 117 | 118 | 940 | 0.5 |
| 10 | 6 | 0 | 16 | 15 | 78 | 106 | 135 | 964 | 0.5 |
| 11 | 6 | 0 | 16.5 | 16 | 78 | 119 | 138 | 1028 | 0.5 |
| 12 | 6 | 0 | 17.5 | 17 | 76 | 114 | 140 | 1016 | 0.5 |
| 13 | 6 | 0 | 14.5 | 14.5 | 77 | 119 | 141 | 1040 | 0.5 |
| 14 | 6 | 0 | 15.5 | 15 | 79 | 121 | 128 | 996 | 0.5 |
| 15 | 6 | 0 | 16.5 | 16 | 79 | 126 | 128 | 1016 | 0.5 |
| 16 | 6 | 0 | 16.5 | 15.5 | 78 | 111 | 134 | 980 | 0.5 |
| 17 | 6 | 0 | 16 | 15.5 | 78 | 118 | 135 | 1012 | 0.5 |
| 18 | 6 | 0 | 17 | 16.5 | 80 | 132 | 130 | 1048 | 0.5 |
| 19 | 6 | 0 | 16 | 15.5 | 79 | 124 | 136 | 1040 | 0.5 |
| 20 | 6 | 0 | 16.5 | 14.5 | 78 | 93 | 132 | 900 | 0.5 |
| 1 | 1 | 1 | 21 | 14 | 70 | -42 | 160 | 472 | 0.25 |
| 2 | 1 | 1 | 21 | 13 | 65 | -63 | 161 | 392 | 0.25 |
| 3 | 1 | 1 | 18 | 16 | 78 | 92 | 145 | 948 | 0.25 |
| 4 | 1 | 1 | 19 | 16 | 70 | 44 | 160 | 816 | 0.25 |
| 5 | 1 | 1 | 19 | 15 | 65 | 9 | 188 | 788 | 0.25 |
| 6 | 1 | 1 | 19 | 15 | 67 | 19 | 162 | 724 | 0.25 |
| 7 | 1 | 1 | 17 | 14 | 65 | 15 | 177 | 768 | 0.25 |
| 8 | 1 | 1 | 18 | 13 | 67 | -5 | 148 | 572 | 0.25 |
| 9 | 1 | 1 | 18 | 12 | 69 | -10 | 108 | 392 | 0.25 |
| 10 | 1 | 1 | 18 | 14 | 72 | 32 | 147 | 716 | 0.25 |
| 11 | 1 | 1 | 21 | 16 | 66 | -15 | 190 | 700 | 0.25 |
| 12 | 1 | 1 | 16 | 13 | 72 | 54 | 136 | 760 | 0.25 |
| 13 | 1 | 1 | 14 | 9 | 71 | 10 | 115 | 500 | 0.25 |
| 14 | 1 | 1 | 16 | 12 | 72 | 35 | 134 | 676 | 0.25 |
| 15 | 1 | 1 | 15 | 10 | 74 | 21 | 113 | 536 | 0.25 |
| 16 | 1 | 1 | 14 | 10 | 68 | 21 | 133 | 616 | 0.25 |
| 17 | 1 | 1 | 15 | 11 | 77 | 48 | 111 | 636 | 0.25 |
| 18 | 1 | 1 | 15 | 9 | 65 | -16 | 119 | 412 | 0.25 |
| 19 | 1 | 1 | 14 | 10 | 73 | 34 | 115 | 596 | 0.25 |
| 20 | 1 | 1 | 14 | 11 | 74 | 55 | 111 | 664 | 0.25 |
| 1 | 2 | 1 | 16 | 13 | 77 | 71 | 128 | 796 | 0.44 |
| 2 | 2 | 1 | 19 | 15 | 77 | 54 | 130 | 736 | 0.44 |
| 3 | 2 | 1 | 18 | 13 | 83 | 57 | 110 | 668 | 0.44 |
| 4 | 2 | 1 | 17 | 14 | 83 | 94 | 117 | 844 | 0.44 |
| 5 | 2 | 1 | 20 | 16 | 81 | 70 | 124 | 776 | 0.44 |
| 6 | 2 | 1 | 20 | 18 | 83 | 119 | 116 | 940 | 0.44 |
| 7 | 2 | 1 | 21 | 17 | 86 | 85 | 110 | 780 | 0.44 |
| 8 | 2 | 1 | 18 | 13 | 83 | 54 | 112 | 664 | 0.44 |
| 9 | 2 | 1 | 19 | 15 | 83 | 78 | 99 | 708 | 0.44 |
| 10 | 2 | 1 | 18 | 16 | 85 | 123 | 111 | 936 | 0.44 |
| 11 | 2 | 1 | 19 | 17 | 82 | 110 | 124 | 936 | 0.44 |
| 12 | 2 | 1 | 18 | 14 | 83 | 75 | 112 | 748 | 0.44 |
| 13 | 2 | 1 | 18 | 13 | 80 | 43 | 121 | 656 | 0.44 |
| 14 | 2 | 1 | 20 | 18 | 80 | 95 | 124 | 876 | 0.44 |
| 15 | 2 | 1 | 17 | 13 | 80 | 65 | 117 | 728 | 0.44 |
| 16 | 2 | 1 | 18 | 15 | 82 | 91 | 121 | 848 | 0.44 |
| 17 | 2 | 1 | 18 | 13 | 84 | 58 | 106 | 656 | 0.44 |
| 18 | 2 | 1 | 17 | 12 | 82 | 51 | 103 | 616 | 0.44 |
| 19 | 2 | 1 | 18 | 14 | 84 | 78 | 108 | 744 | 0.44 |
| 20 | 2 | 1 | 17 | 12 | 86 | 61 | 91 | 608 | 0.44 |
| 1 | 3 | 1 | 15 | 13 | 79 | 89 | 118 | 828 | 0.72 |
| 2 | 3 | 1 | 18 | 16 | 80 | 105 | 137 | 968 | 0.72 |
| 3 | 3 | 1 | 18 | 12 | 85 | 42 | 94 | 544 | 0.72 |
| 4 | 3 | 1 | 18 | 12 | 78 | 20 | 122 | 568 | 0.72 |
| 5 | 3 | 1 | 18 | 11 | 93 | 42 | 68 | 440 | 0.72 |
| 6 | 3 | 1 | 19 | 16 | 85 | 108 | 114 | 888 | 0.72 |
| 7 | 3 | 1 | 19 | 16 | 86 | 106 | 105 | 844 | 0.72 |
| 8 | 3 | 1 | 18 | 14 | 84 | 79 | 101 | 720 | 0.72 |
| 9 | 3 | 1 | 18 | 15 | 83 | 97 | 117 | 856 | 0.72 |
| 10 | 3 | 1 | 19 | 15 | 86 | 90 | 107 | 788 | 0.72 |
| 11 | 3 | 1 | 18 | 16 | 86 | 132 | 100 | 928 | 0.72 |
| 12 | 3 | 1 | 18 | 13 | 88 | 72 | 93 | 660 | 0.72 |
| 13 | 3 | 1 | 18 | 13 | 84 | 61 | 101 | 648 | 0.72 |
| 14 | 3 | 1 | 19 | 13 | 85 | 43 | 101 | 576 | 0.72 |
| 15 | 3 | 1 | 19 | 14 | 84 | 61 | 113 | 696 | 0.72 |
| 16 | 3 | 1 | 19 | 16 | 81 | 91 | 108 | 796 | 0.72 |
| 17 | 3 | 1 | 18 | 14 | 86 | 87 | 104 | 764 | 0.72 |
| 18 | 3 | 1 | 18 | 16 | 86 | 131 | 111 | 968 | 0.72 |
| 19 | 3 | 1 | 18 | 10 | 90 | 10 | 77 | 348 | 0.72 |
| 20 | 3 | 1 | 19 | 15 | 84 | 82 | 117 | 796 | 0.72 |
| 1 | 4 | 1 | 16 | 15 | 70 | 83 | 169 | 1008 | 0.33 |
| 2 | 4 | 1 | 14 | 13 | 70 | 78 | 146 | 896 | 0.33 |
| 3 | 4 | 1 | 17 | 12 | 74 | 23 | 129 | 608 | 0.33 |
| 4 | 4 | 1 | 17 | 13 | 80 | 60 | 114 | 696 | 0.33 |
| 5 | 4 | 1 | 15 | 11 | 76 | 44 | 101 | 580 | 0.33 |
| 6 | 4 | 1 | 15 | 12 | 82 | 80 | 99 | 716 | 0.33 |
| 7 | 4 | 1 | 15 | 10 | 80 | 37 | 102 | 556 | 0.33 |
| 8 | 4 | 1 | 15 | 14 | 75 | 77 | 140 | 868 | 0.33 |
| 9 | 4 | 1 | 14 | 12 | 74 | 73 | 134 | 828 | 0.33 |
| 10 | 4 | 1 | 13 | 11 | 79 | 83 | 111 | 776 | 0.33 |
| 11 | 4 | 1 | 15 | 12 | 77 | 64 | 126 | 760 | 0.33 |
| 12 | 4 | 1 | 16 | 14 | 78 | 91 | 136 | 908 | 0.33 |
| 13 | 4 | 1 | 14 | 11 | 78 | 65 | 100 | 660 | 0.33 |
| 14 | 4 | 1 | 15 | 14 | 76 | 101 | 131 | 928 | 0.33 |
| 15 | 4 | 1 | 14 | 10 | 82 | 52 | 99 | 604 | 0.33 |
| 16 | 4 | 1 | 14 | 13 | 77 | 100 | 114 | 856 | 0.33 |
| 17 | 4 | 1 | 15 | 11 | 84 | 64 | 96 | 640 | 0.33 |
| 18 | 4 | 1 | 16 | 11 | 75 | 19 | 120 | 556 | 0.33 |
| 19 | 4 | 1 | 14 | 12 | 78 | 82 | 123 | 820 | 0.33 |
| 20 | 4 | 1 | 14 | 12 | 80 | 87 | 112 | 796 | 0.33 |
| 1 | 5 | 1 | 16 | 15 | 73 | 91 | 151 | 968 | 0.57 |
| 2 | 5 | 1 | 16 | 12 | 81 | 61 | 101 | 648 | 0.57 |
| 3 | 5 | 1 | 16 | 10 | 73 | 1 | 111 | 448 | 0.57 |
| 4 | 5 | 1 | 15 | 14 | 78 | 87 | 125 | 848 | 0.57 |
| 5 | 5 | 1 | 16 | 12 | 76 | 47 | 127 | 696 | 0.57 |
| 6 | 5 | 1 | 17 | 15 | 82 | 110 | 115 | 900 | 0.57 |
| 7 | 5 | 1 | 17 | 12 | 73 | 24 | 130 | 616 | 0.57 |
| 8 | 5 | 1 | 17 | 13 | 82 | 69 | 113 | 728 | 0.57 |
| 9 | 5 | 1 | 16 | 8 | 75 | -32 | 97 | 260 | 0.57 |
| 10 | 5 | 1 | 16 | 13 | 67 | 38 | 164 | 808 | 0.57 |
| 11 | 5 | 1 | 16 | 12 | 80 | 59 | 110 | 676 | 0.57 |
| 12 | 5 | 1 | 14 | 10 | 80 | 52 | 103 | 620 | 0.57 |
| 13 | 5 | 1 | 14 | 12 | 74 | 73 | 129 | 808 | 0.57 |
| 14 | 5 | 1 | 16 | 14 | 81 | 102 | 120 | 888 | 0.57 |
| 15 | 5 | 1 | 16 | 14 | 78 | 88 | 134 | 888 | 0.57 |
| 16 | 5 | 1 | 16 | 13 | 75 | 61 | 140 | 804 | 0.57 |
| 17 | 5 | 1 | 15 | 12 | 75 | 58 | 131 | 756 | 0.57 |
| 18 | 5 | 1 | 16 | 12 | 80 | 60 | 114 | 696 | 0.57 |
| 19 | 5 | 1 | 15 | 11 | 80 | 54 | 108 | 648 | 0.57 |
| 20 | 5 | 1 | 15 | 6 | 80 | -44 | 64 | 80 | 0.57 |
| 1 | 6 | 1 | 20 | 13 | 73 | -27 | 142 | 460 | 0.25 |
| 2 | 6 | 1 | 20 | 13 | 72 | -29 | 142 | 452 | 0.25 |
| 3 | 6 | 1 | 15 | 13 | 77 | 85 | 114 | 796 | 0.25 |
| 4 | 6 | 1 | 15 | 13 | 79 | 87 | 127 | 856 | 0.25 |
| 5 | 6 | 1 | 15 | 13 | 89 | 126 | 84 | 840 | 0.25 |
| 6 | 6 | 1 | 14 | 8 | 92 | 35 | 62 | 388 | 0.25 |
| 7 | 6 | 1 | 16 | 13 | 78 | 75 | 127 | 808 | 0.25 |
| 8 | 6 | 1 | 15 | 12 | 80 | 75 | 112 | 748 | 0.25 |
| 9 | 6 | 1 | 16 | 11 | 89 | 62 | 87 | 596 | 0.25 |
| 10 | 6 | 1 | 16 | 13 | 76 | 66 | 135 | 804 | 0.25 |
| 11 | 6 | 1 | 15 | 10 | 87 | 50 | 75 | 500 | 0.25 |
| 12 | 6 | 1 | 14 | 12 | 80 | 90 | 105 | 780 | 0.25 |
| 13 | 6 | 1 | 14 | 12 | 76 | 79 | 130 | 836 | 0.25 |
| 14 | 6 | 1 | 15 | 11 | 80 | 55 | 92 | 588 | 0.25 |
| 15 | 6 | 1 | 13 | 8 | 82 | 29 | 83 | 448 | 0.25 |
| 16 | 6 | 1 | 17 | 13 | 77 | 53 | 129 | 728 | 0.25 |
| 17 | 6 | 1 | 15 | 13 | 80 | 95 | 117 | 848 | 0.25 |
| 18 | 6 | 1 | 14 | 12 | 82 | 98 | 108 | 824 | 0.25 |
| 19 | 6 | 1 | 14 | 9 | 70 | 8 | 113 | 484 | 0.25 |
| 20 | 6 | 1 | 15 | 11 | 74 | 37 | 115 | 608 | 0.25 |
| 1 | 1 | 2 | 13 | 12 | 52 | -54 | 129 | 300 | 0.5 |
| 2 | 1 | 2 | 15 | 14 | 61 | 0 | 160 | 640 | 0.5 |
| 3 | 1 | 2 | 12 | 12 | 59 | 39 | 160 | 796 | 0.5 |
| 4 | 1 | 2 | 18 | 14 | 66 | -104 | 111 | 28 | 0.5 |
| 5 | 1 | 2 | 18 | 17 | 62 | -46 | 153 | 428 | 0.5 |
| 6 | 1 | 2 | 15 | 15 | 65 | -37 | 110 | 292 | 0.5 |
| 7 | 1 | 2 | 14 | 13 | 66 | 47 | 155 | 808 | 0.5 |
| 8 | 1 | 2 | 15 | 15 | 70 | 51 | 121 | 688 | 0.5 |
| 9 | 1 | 2 | 15 | 14 | 65 | 19 | 133 | 608 | 0.5 |
| 10 | 1 | 2 | 17 | 15 | 63 | -31 | 144 | 452 | 0.5 |
| 11 | 1 | 2 | 12 | 12 | 66 | 40 | 130 | 680 | 0.5 |
| 12 | 1 | 2 | 11 | 11 | 65 | 17 | 114 | 524 | 0.5 |
| 13 | 1 | 2 | 11 | 10 | 62 | 0 | 104 | 416 | 0.5 |
| 14 | 1 | 2 | 11 | 11 | 62 | 30 | 131 | 644 | 0.5 |
| 15 | 1 | 2 | 12 | 12 | 58 | 3 | 144 | 588 | 0.5 |
| 16 | 1 | 2 | 11 | 11 | 63 | 53 | 142 | 780 | 0.5 |
| 17 | 1 | 2 | 11 | 11 | 55 | 25 | 167 | 768 | 0.5 |
| 18 | 1 | 2 | 11 | 10 | 61 | 30 | 132 | 648 | 0.5 |
| 19 | 1 | 2 | 11 | 11 | 67 | 27 | 98 | 500 | 0.5 |
| 20 | 1 | 2 | 11 | 11 | 62 | 14 | 121 | 540 | 0.5 |
| 1 | 2 | 2 | 17 | 13 | 73 | -56 | 88 | 128 | 0.5 |
| 2 | 2 | 2 | 16 | 14 | 73 | 33 | 96 | 516 | 0.5 |
| 3 | 2 | 2 | 14 | 13 | 76 | 69 | 127 | 784 | 0.5 |
| 4 | 2 | 2 | 21 | 15 | 70 | -47 | 139 | 368 | 0.5 |
| 5 | 2 | 2 | 17 | 17 | 70 | 37 | 163 | 800 | 0.5 |
| 6 | 2 | 2 | 23 | 22 | 62 | -33 | 193 | 640 | 0.5 |
| 7 | 2 | 2 | 15 | 15 | 63 | -54 | 131 | 308 | 0.5 |
| 8 | 2 | 2 | 14 | 14 | 62 | -9 | 132 | 492 | 0.5 |
| 9 | 2 | 2 | 14 | 11 | 59 | -4 | 149 | 580 | 0.5 |
| 10 | 2 | 2 | 15 | 14 | 56 | -67 | 112 | 180 | 0.5 |
| 11 | 2 | 2 | 12 | 12 | 55 | 5 | 157 | 648 | 0.5 |
| 12 | 2 | 2 | 11 | 11 | 56 | -30 | 105 | 300 | 0.5 |
| 13 | 2 | 2 | 10 | 8 | 52 | -2 | 137 | 540 | 0.5 |
| 14 | 2 | 2 | 9 | 9 | 59 | 5 | 94 | 396 | 0.5 |
| 15 | 2 | 2 | 11 | 11 | 68 | 6 | 59 | 260 | 0.5 |
| 16 | 2 | 2 | 11 | 11 | 52 | -16 | 106 | 360 | 0.5 |
| 17 | 2 | 2 | 8 | 8 | 62 | 51 | 123 | 696 | 0.5 |
| 18 | 2 | 2 | 8 | 8 | 64 | 10 | 72 | 328 | 0.5 |
| 19 | 2 | 2 | 8 | 8 | 64 | 13 | 88 | 404 | 0.5 |
| 20 | 2 | 2 | 7 | 7 | 65 | 17 | 74 | 364 | 0.5 |
| 1 | 3 | 2 | 20 | 16 | 66 | -1 | 178 | 708 | 0.625 |
| 2 | 3 | 2 | 16 | 16 | 65 | 28 | 123 | 604 | 0.625 |
| 3 | 3 | 2 | 16 | 14 | 65 | -1 | 136 | 540 | 0.625 |
| 4 | 3 | 2 | 17 | 14 | 68 | -23 | 125 | 408 | 0.625 |
| 5 | 3 | 2 | 15 | 14 | 70 | 14 | 100 | 456 | 0.625 |
| 6 | 3 | 2 | 13 | 13 | 73 | 63 | 94 | 628 | 0.625 |
| 7 | 3 | 2 | 13 | 13 | 74 | 48 | 74 | 488 | 0.625 |
| 8 | 3 | 2 | 13 | 13 | 78 | 95 | 91 | 744 | 0.625 |
| 9 | 3 | 2 | 14 | 14 | 74 | 49 | 130 | 716 | 0.625 |
| 10 | 3 | 2 | 13 | 13 | 79 | 72 | 74 | 584 | 0.625 |
| 11 | 3 | 2 | 15 | 15 | 76 | 51 | 91 | 568 | 0.625 |
| 12 | 3 | 2 | 14 | 14 | 78 | 96 | 109 | 820 | 0.625 |
| 13 | 3 | 2 | 13 | 13 | 80 | 63 | 91 | 616 | 0.625 |
| 14 | 3 | 2 | 14 | 14 | 80 | 90 | 92 | 728 | 0.625 |
| 15 | 3 | 2 | 14 | 14 | 79 | 52 | 75 | 508 | 0.625 |
| 16 | 3 | 2 | 14 | 14 | 76 | 52 | 110 | 648 | 0.625 |
| 17 | 3 | 2 | 14 | 14 | 79 | 88 | 86 | 696 | 0.625 |
| 18 | 3 | 2 | 13 | 13 | 82 | 4 | 52 | 224 | 0.625 |
| 19 | 3 | 2 | 13 | 12 | 87 | 108 | 68 | 704 | 0.625 |
| 20 | 3 | 2 | 14 | 14 | 81 | 70 | 102 | 688 | 0.625 |
| 1 | 4 | 2 | 14 | 13 | 84 | 68 | 71 | 556 | 0.625 |
| 2 | 4 | 2 | 14 | 14 | 91 | 71 | 63 | 536 | 0.625 |
| 3 | 4 | 2 | 14 | 14 | 101 | 133 | 37 | 680 | 0.625 |
| 4 | 4 | 2 | 15 | 15 | 91 | 111 | 56 | 668 | 0.625 |
| 5 | 4 | 2 | 16 | 16 | 91 | 103 | 49 | 608 | 0.625 |
| 6 | 4 | 2 | 20 | 19 | 87 | 41 | 71 | 448 | 0.625 |
| 7 | 4 | 2 | 16 | 16 | 93 | 152 | 63 | 860 | 0.625 |
| 8 | 4 | 2 | 16 | 16 | 98 | 184 | 47 | 924 | 0.625 |
| 9 | 4 | 2 | 17 | 16 | 91 | 68 | 69 | 548 | 0.625 |
| 10 | 4 | 2 | 17 | 16 | 88 | 90 | 65 | 620 | 0.625 |
| 11 | 4 | 2 | 17 | 17 | 87 | 91 | 65 | 624 | 0.625 |
| 12 | 4 | 2 | 16 | 16 | 91 | 117 | 59 | 704 | 0.625 |
| 13 | 4 | 2 | 18 | 17 | 80 | 83 | 86 | 676 | 0.625 |
| 14 | 4 | 2 | 19 | 18 | 82 | 41 | 91 | 528 | 0.625 |
| 15 | 4 | 2 | 17 | 17 | 92 | 127 | 75 | 808 | 0.625 |
| 16 | 4 | 2 | 16 | 16 | 90 | 71 | 54 | 500 | 0.625 |
| 17 | 4 | 2 | 16 | 16 | 89 | 113 | 69 | 728 | 0.625 |
| 18 | 4 | 2 | 18 | 17 | 90 | 62 | 62 | 496 | 0.625 |
| 19 | 4 | 2 | 17 | 17 | 91 | 165 | 89 | 1016 | 0.625 |
| 20 | 4 | 2 | 15 | 15 | 91 | 132 | 83 | 860 | 0.625 |
| 1 | 5 | 2 | 12 | 12 | 82 | 104 | 92 | 784 | 0.75 |
| 2 | 5 | 2 | 12 | 12 | 78 | 18 | 61 | 316 | 0.75 |
| 3 | 5 | 2 | 13 | 13 | 82 | 72 | 75 | 588 | 0.75 |
| 4 | 5 | 2 | 14 | 14 | 75 | 16 | 103 | 476 | 0.75 |
| 5 | 5 | 2 | 14 | 14 | 76 | 97 | 135 | 928 | 0.75 |
| 6 | 5 | 2 | 13 | 12 | 74 | 38 | 89 | 508 | 0.75 |
| 7 | 5 | 2 | 13 | 13 | 84 | 32 | 68 | 400 | 0.75 |
| 8 | 5 | 2 | 11 | 11 | 84 | 97 | 80 | 708 | 0.75 |
| 9 | 5 | 2 | 13 | 12 | 82 | 83 | 81 | 656 | 0.75 |
| 10 | 5 | 2 | 11 | 11 | 82 | 60 | 71 | 524 | 0.75 |
| 11 | 5 | 2 | 11 | 11 | 86 | 92 | 70 | 648 | 0.75 |
| 12 | 5 | 2 | 11 | 11 | 89 | 115 | 72 | 748 | 0.75 |
| 13 | 5 | 2 | 10 | 10 | 87 | 56 | 53 | 436 | 0.75 |
| 14 | 5 | 2 | 10 | 10 | 85 | 69 | 67 | 544 | 0.75 |
| 15 | 5 | 2 | 11 | 11 | 80 | 81 | 73 | 616 | 0.75 |
| 16 | 5 | 2 | 10 | 10 | 85 | 63 | 56 | 476 | 0.75 |
| 17 | 5 | 2 | 11 | 11 | 88 | 29 | 47 | 304 | 0.75 |
| 18 | 5 | 2 | 10 | 10 | 85 | 118 | 88 | 824 | 0.75 |
| 19 | 5 | 2 | 10 | 10 | 83 | 22 | 59 | 324 | 0.75 |
| 20 | 5 | 2 | 10 | 10 | 89 | 108 | 63 | 684 | 0.75 |
| 1 | 6 | 2 | 12 | 11 | 57 | -41 | 93 | 208 | 0.125 |
| 2 | 6 | 2 | 14 | 13 | 50 | -24 | 164 | 560 | 0.125 |
| 3 | 6 | 2 | 11 | 11 | 59 | 2 | 127 | 516 | 0.125 |
| 4 | 6 | 2 | 13 | 13 | 54 | -36 | 139 | 412 | 0.125 |
| 5 | 6 | 2 | 13 | 12 | 56 | -28 | 126 | 392 | 0.125 |
| 6 | 6 | 2 | 16 | 13 | 56 | -50 | 128 | 312 | 0.125 |
| 7 | 6 | 2 | 13 | 13 | 56 | 21 | 161 | 728 | 0.125 |
| 8 | 6 | 2 | 17 | 14 | 59 | -24 | 159 | 540 | 0.125 |
| 9 | 6 | 2 | 15 | 14 | 57 | 19 | 198 | 868 | 0.125 |
| 10 | 6 | 2 | 12 | 12 | 59 | 4 | 102 | 424 | 0.125 |
| 11 | 6 | 2 | 14 | 14 | 58 | 8 | 167 | 700 | 0.125 |
| 12 | 6 | 2 | 15 | 14 | 59 | -15 | 118 | 412 | 0.125 |
| 13 | 6 | 2 | 13 | 12 | 60 | -9 | 149 | 560 | 0.125 |
| 14 | 6 | 2 | 12 | 11 | 62 | 6 | 114 | 480 | 0.125 |
| 15 | 6 | 2 | 13 | 13 | 64 | 24 | 140 | 656 | 0.125 |
| 16 | 6 | 2 | 12 | 12 | 70 | 90 | 147 | 948 | 0.125 |
| 17 | 6 | 2 | 12 | 11 | 70 | -20 | 80 | 240 | 0.125 |
| 18 | 6 | 2 | 13 | 13 | 66 | 15 | 119 | 536 | 0.125 |
| 19 | 6 | 2 | 13 | 13 | 65 | 1 | 95 | 384 | 0.125 |
| 20 | 6 | 2 | 13 | 12 | 64 | 32 | 129 | 644 | 0.125 |
| 1 | 1 | 4 | 19 | 16 | 64 | 23 | 148 | 684 | 0.375 |
| 2 | 1 | 4 | 16 | 13 | 63 | 25 | 145 | 680 | 0.375 |
| 3 | 1 | 4 | 16 | 13 | 63 | 24 | 133 | 628 | 0.375 |
| 4 | 1 | 4 | 14 | 14 | 66 | 71 | 134 | 820 | 0.375 |
| 5 | 1 | 4 | 17 | 15 | 65 | 46 | 146 | 768 | 0.375 |
| 6 | 1 | 4 | 16 | 13 | 66 | 32 | 136 | 672 | 0.375 |
| 7 | 1 | 4 | 14 | 13 | 65 | 63 | 130 | 772 | 0.375 |
| 8 | 1 | 4 | 14 | 14 | 66 | 83 | 130 | 852 | 0.375 |
| 9 | 1 | 4 | 14 | 14 | 68 | 89 | 133 | 888 | 0.375 |
| 10 | 1 | 4 | 15 | 15 | 68 | 90 | 135 | 900 | 0.375 |
| 11 | 1 | 4 | 15 | 13 | 69 | 59 | 121 | 720 | 0.375 |
| 12 | 1 | 4 | 14 | 14 | 65 | 79 | 136 | 860 | 0.375 |
| 13 | 1 | 4 | 14 | 12 | 62 | 38 | 127 | 660 | 0.375 |
| 14 | 1 | 4 | 14 | 14 | 66 | 84 | 135 | 876 | 0.375 |
| 15 | 1 | 4 | 15 | 15 | 60 | 57 | 158 | 860 | 0.375 |
| 16 | 1 | 4 | 15 | 14 | 63 | 57 | 145 | 808 | 0.375 |
| 17 | 1 | 4 | 14 | 14 | 64 | 75 | 145 | 880 | 0.375 |
| 18 | 1 | 4 | 14 | 12 | 61 | 35 | 132 | 668 | 0.375 |
| 19 | 1 | 4 | 15 | 15 | 65 | 81 | 143 | 896 | 0.375 |
| 20 | 1 | 4 | 15 | 14 | 64 | 59 | 137 | 784 | 0.375 |
| 1 | 2 | 4 | 13 | 13 | 63 | 67 | 142 | 836 | 0.375 |
| 2 | 2 | 4 | 14 | 14 | 65 | 77 | 132 | 836 | 0.375 |
| 3 | 2 | 4 | 14 | 14 | 69 | 87 | 123 | 840 | 0.375 |
| 4 | 2 | 4 | 14 | 14 | 65 | 72 | 134 | 824 | 0.375 |
| 5 | 2 | 4 | 14 | 14 | 64 | 74 | 148 | 888 | 0.375 |
| 6 | 2 | 4 | 13 | 12 | 56 | 32 | 145 | 708 | 0.375 |
| 7 | 2 | 4 | 14 | 13 | 62 | 47 | 129 | 704 | 0.375 |
| 8 | 2 | 4 | 13 | 13 | 68 | 88 | 126 | 856 | 0.375 |
| 9 | 2 | 4 | 13 | 13 | 71 | 97 | 116 | 852 | 0.375 |
| 10 | 2 | 4 | 13 | 13 | 63 | 72 | 135 | 828 | 0.375 |
| 11 | 2 | 4 | 12 | 11 | 67 | 62 | 110 | 688 | 0.375 |
| 12 | 2 | 4 | 14 | 14 | 77 | 117 | 101 | 872 | 0.375 |
| 13 | 2 | 4 | 14 | 14 | 66 | 80 | 116 | 784 | 0.375 |
| 14 | 2 | 4 | 14 | 14 | 67 | 87 | 122 | 836 | 0.375 |
| 15 | 2 | 4 | 13 | 13 | 69 | 89 | 122 | 844 | 0.375 |
| 16 | 2 | 4 | 14 | 14 | 73 | 106 | 110 | 864 | 0.375 |
| 17 | 2 | 4 | 14 | 14 | 73 | 106 | 118 | 896 | 0.375 |
| 18 | 2 | 4 | 14 | 14 | 74 | 112 | 110 | 888 | 0.375 |
| 19 | 2 | 4 | 14 | 13 | 75 | 96 | 104 | 800 | 0.375 |
| 20 | 2 | 4 | 13 | 13 | 74 | 105 | 115 | 880 | 0.375 |
| 1 | 3 | 4 | 20 | 15 | 62 | -24 | 159 | 540 | 0.375 |
| 2 | 3 | 4 | 22 | 17 | 65 | -24 | 159 | 540 | 0.375 |
| 3 | 3 | 4 | 19 | 16 | 68 | 29 | 145 | 696 | 0.375 |
| 4 | 3 | 4 | 16 | 15 | 69 | 79 | 134 | 852 | 0.375 |
| 5 | 3 | 4 | 16 | 16 | 70 | 95 | 136 | 924 | 0.375 |
| 6 | 3 | 4 | 20 | 18 | 65 | 36 | 153 | 756 | 0.375 |
| 7 | 3 | 4 | 17 | 15 | 68 | 53 | 137 | 760 | 0.375 |
| 8 | 3 | 4 | 18 | 16 | 70 | 55 | 146 | 804 | 0.375 |
| 9 | 3 | 4 | 17 | 15 | 69 | 57 | 132 | 756 | 0.375 |
| 10 | 3 | 4 | 17 | 14 | 62 | 7 | 151 | 632 | 0.375 |
| 11 | 3 | 4 | 17 | 15 | 70 | 63 | 127 | 760 | 0.375 |
| 12 | 3 | 4 | 18 | 17 | 68 | 64 | 152 | 864 | 0.375 |
| 13 | 3 | 4 | 14 | 14 | 69 | 94 | 113 | 828 | 0.375 |
| 14 | 3 | 4 | 14 | 14 | 73 | 107 | 123 | 920 | 0.375 |
| 15 | 3 | 4 | 16 | 14 | 72 | 70 | 113 | 732 | 0.375 |
| 16 | 3 | 4 | 16 | 16 | 65 | 76 | 149 | 900 | 0.375 |
| 17 | 3 | 4 | 17 | 15 | 73 | 71 | 123 | 776 | 0.375 |
| 18 | 3 | 4 | 16 | 16 | 71 | 100 | 131 | 924 | 0.375 |
| 19 | 3 | 4 | 15 | 15 | 68 | 90 | 139 | 916 | 0.375 |
| 20 | 3 | 4 | 15 | 13 | 69 | 59 | 114 | 692 | 0.375 |
| 1 | 4 | 4 | 27 | 21 | 68 | -46 | 163 | 468 | 0.5 |
| 2 | 4 | 4 | 19 | 17 | 71 | 70 | 133 | 812 | 0.5 |
| 3 | 4 | 4 | 18 | 18 | 70 | 103 | 146 | 996 | 0.5 |
| 4 | 4 | 4 | 19 | 19 | 72 | 111 | 143 | 1016 | 0.5 |
| 5 | 4 | 4 | 18 | 17 | 71 | 88 | 136 | 896 | 0.5 |
| 6 | 4 | 4 | 16 | 15 | 70 | 80 | 132 | 848 | 0.5 |
| 7 | 4 | 4 | 17 | 15 | 68 | 57 | 142 | 796 | 0.5 |
| 8 | 4 | 4 | 16 | 15 | 71 | 84 | 126 | 840 | 0.5 |
| 9 | 4 | 4 | 17 | 16 | 72 | 88 | 123 | 844 | 0.5 |
| 10 | 4 | 4 | 17 | 15 | 68 | 57 | 137 | 776 | 0.5 |
| 11 | 4 | 4 | 15 | 14 | 74 | 94 | 116 | 840 | 0.5 |
| 12 | 4 | 4 | 14 | 14 | 71 | 100 | 130 | 920 | 0.5 |
| 13 | 4 | 4 | 16 | 15 | 69 | 81 | 141 | 888 | 0.5 |
| 14 | 4 | 4 | 13 | 13 | 76 | 92 | 118 | 840 | 0.5 |
| 15 | 4 | 4 | 14 | 14 | 67 | 84 | 142 | 904 | 0.5 |
| 16 | 4 | 4 | 14 | 10 | 74 | 36 | 96 | 528 | 0.5 |
| 17 | 4 | 4 | 14 | 14 | 80 | 132 | 91 | 892 | 0.5 |
| 18 | 4 | 4 | 14 | 13 | 83 | 121 | 85 | 824 | 0.5 |
| 19 | 4 | 4 | 16 | 16 | 76 | 123 | 108 | 924 | 0.5 |
| 20 | 4 | 4 | 15 | 14 | 78 | 111 | 104 | 860 | 0.5 |
| 1 | 5 | 4 | 15 | 15 | 66 | 80 | 142 | 888 | 0.25 |
| 2 | 5 | 4 | 17 | 17 | 57 | 38 | 185 | 892 | 0.25 |
| 3 | 5 | 4 | 14 | 14 | 75 | 110 | 109 | 876 | 0.25 |
| 4 | 5 | 4 | 15 | 15 | 71 | 99 | 130 | 916 | 0.25 |
| 5 | 5 | 4 | 15 | 15 | 75 | 111 | 117 | 912 | 0.25 |
| 6 | 5 | 4 | 13 | 13 | 82 | 131 | 89 | 880 | 0.25 |
| 7 | 5 | 4 | 15 | 15 | 86 | 153 | 82 | 940 | 0.25 |
| 8 | 5 | 4 | 15 | 15 | 76 | 115 | 116 | 924 | 0.25 |
| 9 | 5 | 4 | 14 | 14 | 81 | 131 | 99 | 920 | 0.25 |
| 10 | 5 | 4 | 14 | 14 | 81 | 133 | 98 | 924 | 0.25 |
| 11 | 5 | 4 | 13 | 13 | 85 | 140 | 84 | 896 | 0.25 |
| 12 | 5 | 4 | 13 | 13 | 77 | 115 | 92 | 828 | 0.25 |
| 13 | 5 | 4 | 13 | 13 | 77 | 113 | 97 | 840 | 0.25 |
| 14 | 5 | 4 | 13 | 13 | 79 | 121 | 95 | 864 | 0.25 |
| 15 | 5 | 4 | 13 | 12 | 76 | 91 | 99 | 760 | 0.25 |
| 16 | 5 | 4 | 12 | 11 | 83 | 108 | 80 | 752 | 0.25 |
| 17 | 5 | 4 | 13 | 13 | 73 | 100 | 107 | 828 | 0.25 |
| 18 | 5 | 4 | 12 | 12 | 78 | 113 | 94 | 828 | 0.25 |
| 19 | 5 | 4 | 13 | 12 | 79 | 100 | 100 | 800 | 0.25 |
| 20 | 5 | 4 | 12 | 12 | 82 | 125 | 88 | 852 | 0.25 |
| 1 | 6 | 4 | 14 | 13 | 72 | 85 | 122 | 828 | 0.125 |
| 2 | 6 | 4 | 17 | 12 | 75 | 22 | 95 | 468 | 0.125 |
| 3 | 6 | 4 | 17 | 15 | 78 | 93 | 100 | 772 | 0.125 |
| 4 | 6 | 4 | 11 | 10 | 80 | 93 | 81 | 696 | 0.125 |
| 5 | 6 | 4 | 14 | 13 | 80 | 107 | 93 | 800 | 0.125 |
| 6 | 6 | 4 | 13 | 12 | 82 | 108 | 84 | 768 | 0.125 |
| 7 | 6 | 4 | 13 | 13 | 80 | 122 | 100 | 888 | 0.125 |
| 8 | 6 | 4 | 15 | 8 | 73 | -20 | 73 | 212 | 0.125 |
| 9 | 6 | 4 | 13 | 12 | 76 | 94 | 99 | 772 | 0.125 |
| 10 | 6 | 4 | 14 | 15 | 74 | 102 | 122 | 896 | 0.125 |
| 11 | 6 | 4 | 13 | 11 | 77 | 73 | 90 | 652 | 0.125 |
| 12 | 6 | 4 | 13 | 13 | 73 | 101 | 113 | 856 | 0.125 |
| 13 | 6 | 4 | 12 | 11 | 72 | 71 | 105 | 704 | 0.125 |
| 14 | 6 | 4 | 16 | 16 | 72 | 106 | 136 | 968 | 0.125 |
| 15 | 6 | 4 | 15 | 12 | 69 | 38 | 105 | 572 | 0.125 |
| 16 | 6 | 4 | 15 | 14 | 69 | 76 | 123 | 796 | 0.125 |
| 17 | 6 | 4 | 13 | 13 | 73 | 104 | 113 | 868 | 0.125 |
| 18 | 6 | 4 | 14 | 14 | 71 | 98 | 125 | 892 | 0.125 |
| 19 | 6 | 4 | 13 | 13 | 72 | 100 | 110 | 840 | 0.125 |
| 20 | 6 | 4 | 15 | 15 | 69 | 96 | 127 | 892 | 0.125 |
| 1 | 1 | 5 | 14 | 14 | 63 | 57 | 149 | 824 | 0.375 |
| 2 | 1 | 5 | 14 | 13 | 68 | 57 | 125 | 728 | 0.375 |
| 3 | 1 | 5 | 14 | 12 | 76 | 58 | 101 | 636 | 0.375 |
| 4 | 1 | 5 | 15 | 14 | 76 | 80 | 112 | 768 | 0.375 |
| 5 | 1 | 5 | 13 | 11 | 76 | 59 | 93 | 608 | 0.375 |
| 6 | 1 | 5 | 12 | 12 | 74 | 81 | 108 | 756 | 0.375 |
| 7 | 1 | 5 | 12 | 12 | 73 | 79 | 109 | 752 | 0.375 |
| 8 | 1 | 5 | 13 | 13 | 78 | 91 | 111 | 808 | 0.375 |
| 9 | 1 | 5 | 12 | 12 | 80 | 94 | 96 | 760 | 0.375 |
| 10 | 1 | 5 | 13 | 8 | 72 | 5 | 79 | 336 | 0.375 |
| 11 | 1 | 5 | 12 | 11 | 77 | 73 | 97 | 680 | 0.375 |
| 12 | 1 | 5 | 12 | 12 | 77 | 92 | 101 | 772 | 0.375 |
| 13 | 1 | 5 | 12 | 12 | 78 | 92 | 91 | 732 | 0.375 |
| 14 | 1 | 5 | 13 | 13 | 77 | 92 | 104 | 784 | 0.375 |
| 15 | 1 | 5 | 14 | 14 | 76 | 91 | 111 | 808 | 0.375 |
| 16 | 1 | 5 | 13 | 13 | 80 | 98 | 97 | 780 | 0.375 |
| 17 | 1 | 5 | 13 | 13 | 79 | 98 | 93 | 764 | 0.375 |
| 18 | 1 | 5 | 15 | 14 | 79 | 83 | 109 | 768 | 0.375 |
| 19 | 1 | 5 | 14 | 14 | 77 | 93 | 108 | 804 | 0.375 |
| 20 | 1 | 5 | 13 | 12 | 76 | 76 | 101 | 708 | 0.375 |
| 1 | 2 | 5 | 20 | 20 | 65 | 53 | 176 | 916 | 0.375 |
| 2 | 2 | 5 | 22 | 22 | 72 | 80 | 158 | 952 | 0.375 |
| 3 | 2 | 5 | 21 | 21 | 70 | 79 | 147 | 904 | 0.375 |
| 4 | 2 | 5 | 24 | 24 | 72 | 79 | 145 | 896 | 0.375 |
| 5 | 2 | 5 | 21 | 21 | 74 | 93 | 146 | 956 | 0.375 |
| 6 | 2 | 5 | 22 | 22 | 73 | 87 | 139 | 904 | 0.375 |
| 7 | 2 | 5 | 21 | 21 | 71 | 76 | 132 | 832 | 0.375 |
| 8 | 2 | 5 | 20 | 20 | 78 | 105 | 116 | 884 | 0.375 |
| 9 | 2 | 5 | 21 | 21 | 78 | 103 | 116 | 876 | 0.375 |
| 10 | 2 | 5 | 20 | 20 | 75 | 92 | 127 | 876 | 0.375 |
| 11 | 2 | 5 | 20 | 20 | 76 | 101 | 126 | 908 | 0.375 |
| 12 | 2 | 5 | 20 | 20 | 75 | 92 | 124 | 864 | 0.375 |
| 13 | 2 | 5 | 19 | 19 | 79 | 107 | 118 | 900 | 0.375 |
| 14 | 2 | 5 | 19 | 19 | 75 | 97 | 116 | 852 | 0.375 |
| 15 | 2 | 5 | 18 | 18 | 75 | 86 | 119 | 820 | 0.375 |
| 16 | 2 | 5 | 18 | 18 | 76 | 89 | 123 | 848 | 0.375 |
| 17 | 2 | 5 | 19 | 19 | 77 | 99 | 129 | 912 | 0.375 |
| 18 | 2 | 5 | 18 | 18 | 78 | 97 | 120 | 868 | 0.375 |
| 19 | 2 | 5 | 18 | 18 | 79 | 102 | 109 | 844 | 0.375 |
| 20 | 2 | 5 | 18 | 18 | 78 | 99 | 111 | 840 | 0.375 |
| 1 | 3 | 5 | 16 | 16 | 68 | 73 | 133 | 824 | 0.375 |
| 2 | 3 | 5 | 16 | 16 | 66 | 60 | 146 | 824 | 0.375 |
| 3 | 3 | 5 | 17 | 17 | 77 | 103 | 109 | 848 | 0.375 |
| 4 | 3 | 5 | 19 | 19 | 69 | 73 | 148 | 884 | 0.375 |
| 5 | 3 | 5 | 16 | 16 | 73 | 87 | 127 | 856 | 0.375 |
| 6 | 3 | 5 | 16 | 16 | 78 | 103 | 102 | 820 | 0.375 |
| 7 | 3 | 5 | 17 | 17 | 73 | 89 | 123 | 848 | 0.375 |
| 8 | 3 | 5 | 15 | 14 | 76 | 78 | 102 | 720 | 0.375 |
| 9 | 3 | 5 | 15 | 15 | 76 | 95 | 96 | 764 | 0.375 |
| 10 | 3 | 5 | 16 | 16 | 80 | 112 | 102 | 856 | 0.375 |
| 11 | 3 | 5 | 16 | 16 | 79 | 107 | 102 | 836 | 0.375 |
| 12 | 3 | 5 | 16 | 16 | 84 | 129 | 99 | 912 | 0.375 |
| 13 | 3 | 5 | 18 | 18 | 77 | 100 | 119 | 876 | 0.375 |
| 14 | 3 | 5 | 16 | 16 | 84 | 125 | 93 | 872 | 0.375 |
| 15 | 3 | 5 | 16 | 16 | 85 | 125 | 96 | 884 | 0.375 |
| 16 | 3 | 5 | 16 | 16 | 85 | 126 | 77 | 812 | 0.375 |
| 17 | 3 | 5 | 17 | 17 | 78 | 107 | 111 | 872 | 0.375 |
| 18 | 3 | 5 | 17 | 16 | 81 | 102 | 108 | 840 | 0.375 |
| 19 | 3 | 5 | 17 | 17 | 83 | 125 | 97 | 888 | 0.375 |
| 20 | 3 | 5 | 17 | 17 | 80 | 112 | 107 | 876 | 0.375 |
| 1 | 4 | 5 | 15 | 14 | 67 | 56 | 132 | 752 | 0.25 |
| 2 | 4 | 5 | 15 | 13 | 68 | 42 | 126 | 672 | 0.25 |
| 3 | 4 | 5 | 17 | 15 | 64 | 34 | 150 | 736 | 0.25 |
| 4 | 4 | 5 | 20 | 14 | 67 | -35 | 132 | 388 | 0.25 |
| 5 | 4 | 5 | 15 | 14 | 64 | 47 | 139 | 744 | 0.25 |
| 6 | 4 | 5 | 13 | 11 | 71 | 47 | 94 | 564 | 0.25 |
| 7 | 4 | 5 | 12 | 11 | 72 | 58 | 98 | 624 | 0.25 |
| 8 | 4 | 5 | 12 | 12 | 72 | 70 | 113 | 732 | 0.25 |
| 9 | 4 | 5 | 14 | 12 | 69 | 43 | 117 | 640 | 0.25 |
| 10 | 4 | 5 | 14 | 13 | 72 | 63 | 107 | 680 | 0.25 |
| 11 | 4 | 5 | 13 | 11 | 72 | 41 | 100 | 564 | 0.25 |
| 12 | 4 | 5 | 13 | 13 | 71 | 75 | 123 | 792 | 0.25 |
| 13 | 4 | 5 | 14 | 13 | 77 | 82 | 105 | 748 | 0.25 |
| 14 | 4 | 5 | 13 | 11 | 77 | 57 | 83 | 560 | 0.25 |
| 15 | 4 | 5 | 13 | 13 | 72 | 83 | 103 | 744 | 0.25 |
| 16 | 4 | 5 | 14 | 14 | 71 | 84 | 124 | 832 | 0.25 |
| 17 | 4 | 5 | 12 | 12 | 70 | 73 | 116 | 756 | 0.25 |
| 18 | 4 | 5 | 12 | 12 | 72 | 75 | 106 | 724 | 0.25 |
| 19 | 4 | 5 | 12 | 12 | 78 | 91 | 94 | 740 | 0.25 |
| 20 | 4 | 5 | 12 | 9 | 73 | 31 | 85 | 464 | 0.25 |
| 1 | 5 | 5 | 18 | 18 | 72 | 89 | 141 | 920 | 0.29 |
| 2 | 5 | 5 | 20 | 19 | 74 | 79 | 135 | 856 | 0.29 |
| 3 | 5 | 5 | 20 | 20 | 78 | 103 | 128 | 924 | 0.29 |
| 4 | 5 | 5 | 21 | 20 | 77 | 90 | 126 | 864 | 0.29 |
| 5 | 5 | 5 | 20 | 18 | 73 | 63 | 131 | 776 | 0.29 |
| 6 | 5 | 5 | 17 | 16 | 75 | 79 | 130 | 836 | 0.29 |
| 7 | 5 | 5 | 20 | 20 | 75 | 82 | 115 | 788 | 0.29 |
| 8 | 5 | 5 | 20 | 19 | 78 | 94 | 115 | 836 | 0.29 |
| 9 | 5 | 5 | 17 | 16 | 78 | 91 | 115 | 824 | 0.29 |
| 10 | 5 | 5 | 18 | 16 | 74 | 64 | 119 | 732 | 0.29 |
| 11 | 5 | 5 | 18 | 16 | 81 | 84 | 103 | 748 | 0.29 |
| 12 | 5 | 5 | 18 | 18 | 80 | 119 | 98 | 868 | 0.29 |
| 13 | 5 | 5 | 18 | 17 | 77 | 91 | 106 | 788 | 0.29 |
| 14 | 5 | 5 | 18 | 17 | 75 | 80 | 114 | 776 | 0.29 |
| 15 | 5 | 5 | 15 | 14 | 81 | 97 | 96 | 772 | 0.29 |
| 16 | 5 | 5 | 18 | 17 | 79 | 95 | 111 | 824 | 0.29 |
| 17 | 5 | 5 | 18 | 18 | 83 | 125 | 104 | 916 | 0.29 |
| 18 | 5 | 5 | 18 | 17 | 80 | 99 | 108 | 828 | 0.29 |
| 19 | 5 | 5 | 18 | 18 | 75 | 101 | 128 | 916 | 0.29 |
| 20 | 5 | 5 | 18 | 17 | 72 | 74 | 128 | 808 | 0.29 |
| 1 | 6 | 5 | 16 | 16 | 72 | 82 | 116 | 792 | 0.43 |
| 2 | 6 | 5 | 17 | 16 | 73 | 77 | 129 | 824 | 0.43 |
| 3 | 6 | 5 | 20 | 20 | 79 | 112 | 121 | 932 | 0.43 |
| 4 | 6 | 5 | 20 | 18 | 83 | 90 | 105 | 780 | 0.43 |
| 5 | 6 | 5 | 19 | 19 | 81 | 122 | 109 | 924 | 0.43 |
| 6 | 6 | 5 | 17 | 16 | 77 | 88 | 109 | 788 | 0.43 |
| 7 | 6 | 5 | 18 | 17 | 77 | 92 | 119 | 844 | 0.43 |
| 8 | 6 | 5 | 17 | 16 | 82 | 102 | 105 | 828 | 0.43 |
| 9 | 6 | 5 | 19 | 18 | 76 | 86 | 127 | 852 | 0.43 |
| 10 | 6 | 5 | 19 | 19 | 76 | 100 | 130 | 920 | 0.43 |
| 11 | 6 | 5 | 17 | 17 | 77 | 105 | 122 | 908 | 0.43 |
| 12 | 6 | 5 | 20 | 20 | 69 | 65 | 152 | 868 | 0.43 |
| 13 | 6 | 5 | 17 | 16 | 73 | 73 | 117 | 760 | 0.43 |
| 14 | 6 | 5 | 21 | 21 | 73 | 92 | 146 | 952 | 0.43 |
| 15 | 6 | 5 | 19 | 19 | 72 | 86 | 143 | 916 | 0.43 |
| 16 | 6 | 5 | 17 | 17 | 75 | 101 | 129 | 920 | 0.43 |
| 17 | 6 | 5 | 17 | 17 | 76 | 104 | 124 | 912 | 0.43 |
| 18 | 6 | 5 | 18 | 18 | 74 | 98 | 135 | 932 | 0.43 |
| 19 | 6 | 5 | 18 | 18 | 74 | 96 | 135 | 924 | 0.43 |
| 20 | 6 | 5 | 18 | 18 | 74 | 93 | 133 | 904 | 0.43 |

**SAS nonparametric code and results for Units Produced**

Since the variable, Units Produced, was found to not be normally distributed and was highly skewed [49], the NPAR1WAY procedure, a nonparametric test was used to determine treatment effects of Units Produced in SAS.

The following is the code used in SAS for the non-parametric analysis for Units Produced:

**“DATA** WILCOXON;

OPTIONS

NODATE PAGESIZE = **100** LINESIZE = **200**;

TITLE 'MONEY LAUNDERING PRODUCTION';

INPUT

TREATMENT REPLICATION UNITS PRODUCED;

CARDS;

* NON-PARAMETRIC TESTS FOR PRODUCTION;

**PROC** **PRINT** NOOBS;

**RUN**;

* WILCOXON RANK-SUM TEST BETWEEN TREATMENT 1(base treatment for nonpar1) AND 2;

**DATA** NON_PARA_TEST12;

SET WILCOXON;

IF TREATMENT <=**2** THEN OUTPUT;

**PROC** **PRINT**;

**PROC** **NPAR1WAY** DATA = NON_PARA_TEST12;

CLASS TREATMENT;

VAR UNITS PRODUCED;

EXACT WILCOXON;

**RUN**;

* WILCOXON RANK-SUM TEST BETWEEN TREATMENT 1 AND 3;

**DATA** NON_PARA_TEST13;

SET WILCOXON;

IF TREATMENT =**1** ! TREATMENT = **3** THEN OUTPUT;

**PROC** **PRINT**;

**PROC** **NPAR1WAY** DATA = NON_PARA_TEST13;

CLASS TREATMENT;

VAR UNITS PRODUCED;

EXACT WILCOXON;

**RUN**;

* WILCOXON RANK-SUM TEST BETWEEN TREATMENT 1 AND 4;

**DATA** NON_PARA_TEST14;

SET WILCOXON;

IF TREATMENT =**1** ! TREATMENT = **4** THEN OUTPUT;

**PROC** **PRINT**;

**PROC** **NPAR1WAY** DATA = NON_PARA_TEST14;

CLASS TREATMENT;

VAR UNITS PRODUCED;

EXACT WILCOXON;

**RUN**;

* WILCOXON RANK-SUM TEST BETWEEN TREATMENT 1 AND 5;

**DATA** NON_PARA_TEST15;

SET WILCOXON;

IF TREATMENT =**1** ! TREATMENT = **5** THEN OUTPUT;

**PROC** **PRINT**;

**PROC** **NPAR1WAY** DATA = NON_PARA_TEST15;

CLASS TREATMENT;

VAR UNITS PRODUCED;

EXACT WILCOXON;

**RUN**;

*/ WILCOXON RANK-SUM TEST BETWEEN TREATMENT 1 AND 6;

**DATA** NON_PARA_TEST16;

SET WILCOXON;

IF TREATMENT =**1** ! TREATMENT = **6** THEN OUTPUT;

**PROC** **PRINT**;

**PROC** **NPAR1WAY** DATA = NON_PARA_TEST16;

CLASS TREATMENT;

VAR UNITS PRODUCED;

EXACT WILCOXON;

**RUN**; */”

The following is the resulting output from SAS:

| Wilcoxon Scores (Rank Sums) for Variable Units Produced Classified by Variable TREATMENT  Average scores were used for ties. | | | | | |
| --- | --- | --- | --- | --- | --- |
| TREATMENT | N | Sum of Scores | Expected Under H0 | Std Dev Under H0 | Mean Score |
| No Seizure | 6 | 37.0 | 39.0 | 6.244998 | 6.166667 |
| Product Seizure | 6 | 41.0 | 39.0 | 6.244998 | 6.833333 |

|  | |
| --- | --- |
| Exact Test |  |
| One-Sided Pr <= S | 0.4091 |
| Two-Sided Pr >= \|S - Mean\| | 0.8182 |

| TREATMENT | N | Sum of Scores | Expected Under H0 | Std Dev Under H0 | Mean Score |
| --- | --- | --- | --- | --- | --- |
| No Seizure | 6 | 51.50 | 39.0 | 6.234071 | 8.583333 |
| Trade Seizure | 6 | 26.50 | 39.0 | 6.234071 | 4.416667 |

| Exact Test |  |
| --- | --- |
| One-Sided Pr >= S | 0.0216 |
| Two-Sided Pr >= \|S - Mean\| | 0.0433 |

|  | | | | | |
| --- | --- | --- | --- | --- | --- |
| TREATMENT | N | Sum of Scores | Expected Under H0 | Std Dev Under H0 | Mean Score |
| No Seizure | 6 | 41.50 | 39.0 | 6.234071 | 6.916667 |
| Seller Profit Seizure | 6 | 36.50 | 39.0 | 6.234071 | 6.083333 |
|  | | | | | |

| Exact Test |  |
| --- | --- |
| One-Sided Pr >= S |  |
| Two-Sided Pr >= \|S - Mean\| | 0.3669 |

| TREATMENT | N | Sum of Scores | | Expected Under H0 | | Std Dev Under H0 | Mean Score |
| --- | --- | --- | --- | --- | --- | --- | --- |
| No Seizure | 6 | 33.0 | | 39.0 | | 6.244998 | 5.50 |
| Both Profit Seizure | 6 | 45.0 | | 39.0 | | 6.244998 | 7.50 |
|  | | |  | |  |  |  |
| Exact Test | | |  | |  |  |  |
| One-Sided Pr <= S | | | 0.1970 | |  |  |  |
| Two-Sided Pr >= \|S - Mean\| | | | 0.3939 | |  |  |  |

| TREATMENT | N | Sum of Scores | | Expected Under H0 | | Std Dev Under H0 | Mean Score |
| --- | --- | --- | --- | --- | --- | --- | --- |
| No Seizure | 6 | 47.50 | | 39.0 | | 6.223124 | 7.916667 |
| Buyer Profit Seizure | 6 | 30.50 | | 39.0 | | 6.223124 | 5.083333 |
| Exact Test | | |  | |  |  |  |
| One-Sided Pr >= S | | | 0.0985 | |  |  |  |
| Two-Sided Pr >= \|S - Mean\| | | | 0.1970 | |  |  |  |

## **SAS code for Units Traded**

The PANEL Procedure Parks Method Estimation was conducted for Units Traded in SAS. The following is the SAS code used for the variable Units Traded:

**“DATA** CONVERGENCE;

OPTIONS

NODATE PAGESIZE = **100** LINESIZE = **200**;

TITLE 'MONEY LAUNDERING ANALYSIS';

*All treatment data are averages over 6 replications

Variable Definitions:

TREATMENT is the treatments

0 = Base

1 = Product Seizure

2 = Trade Seizure

3 = Seller Profit Seizure

4 = Buyer Profit Seizure

5 = Both Profit Seizure

PERIOD is the trading period

PRICE is the average market price per trading period

PRODN is the total units produced per period

TRADE is the total units traded per period

SELLEARN is average seller earnings per period

BUYEARN is average buyer earnings per period

TOTEARN is the total earnings per period

PL is the dummy for Production Loss treatment

TL is the dummy for Trade Loss treatment

SPL is the dummy for Seller Profit Loss treatment

BPL is the dummy for Both Profit Loss treatment

YPL is the dummy for Buyer Profit Loss treatment

* Base is NS (No Seizure Treatment);

INPUT

TREATMENT PERIOD PRICE PRODN TRADE SELLEARN BUYEARN TOTEARN PL TL SPL BPL YPL;

CARDS;

..

(note data for this analysis are averages across sessions or replications of data reported above, we do not redundantly report the data here)

..

;

**PROC** **MEANS** DATA = CONVERGENCE;

CLASS TREATMENT;

VAR PRICE PRODN TRADE SELLEARN BUYEARN TOTEARN;

*PANEL PROCEDURE;

**DATA** TWO;

SET CONVERGENCE;

*VARIABLE DEFINITIONS;

ASYM = (PERIOD - **1**)/PERIOD;

SL = **1**/PERIOD;

PLASYM = PL*ASYM;

TLASYM = TL*ASYM;

SPLASYM = SPL*ASYM;

BPLASYM = BPL*ASYM;

YPLASYM = YPL*ASYM;

* DUMMY VARIABLES;

PLSL = PL*SL;

TLSL = TL*SL;

SPLSL = SPL*SL;

BPLSL = BPL*SL;

YPLSL = YPL*SL;

*ASYM (ASYMPTOTE)and SL (STARTING LEVEL) are NS Base Treatment;

**PROC** **PANEL** DATA=TWO;

ID TREATMENT PERIOD;

MODEL TRADE = ASYM PLASYM TLASYM SPLASYM BPLASYM YPLASYM

SL PLSL TLSL SPLSL BPLSL YPLSL / PARKS NOINT;

OUTPUT OUT = RESULT P = PREDICTED R = RESIDUAL;

**PROC** **UNIVARIATE** NORMAL PLOT;

VAR RESIDUAL;

**RUN**;”

The results from this SAS code are as follows:

Dependent Variable: Units Traded

| Model Description | |
| --- | --- |
| Estimation Method | Parks |
| Number of Cross Sections | 6 |
| Time Series Length | 20 |

| Fit Statistics | | | |
| --- | --- | --- | --- |
| SSE | 47.2610 | DFE | 108 |
| MSE | 0.4376 | Root MSE | 0.6615 |
| R-Square | 0.9986 |  |  |

| Parameter Estimates | | | | | |
| --- | --- | --- | --- | --- | --- |
| Variable | DF | Estimate | Standard Error | t Value | Pr > \|t\| |
| No Seizure | 1 | 14.66174 | 0.1632 | 89.82 | <.0001 |
| Product Seizure | 1 | -2.15689 | 0.4455 | -4.84 | <.0001 |
| Trade Seizure | 1 | -4.6413 | 0.7358 | -6.31 | <.0001 |
| Seller Profit Seizure | 1 | 0.366944 | 0.3548 | 1.03 | 0.3034 |
| Both Profit Seizure | 1 | 1.23277 | 0.4357 | 2.83 | 0.0056 |
| Buyer Profit Seizure | 1 | -1.05364 | 0.2498 | -4.22 | <.0001 |
|  |  |  |  |  |  |
|  |  |  |  |  |  |

## **SAS code for Seller Earnings**

The PANEL Procedure Parks Method Estimation was conducted for Seller Earning in SAS. The following is the SAS code used for the variable Seller Earnings:

**“PROC** **PANEL**;

ID TREATMENT PERIOD;

MODEL SELLEARN = ASYM PLASYM TLASYM SPLASYM BPLASYM YPLASYM SL PLSL TLSL SPLSL BPLSL YPLSL / PARKS NOINT;

OUTPUT OUT = RESULT P = PREDICTED R = RESIDUAL;

**PROC** **UNIVARIATE** NORMAL PLOT;

VAR RESIDUAL;

**RUN**;”

Below is the resulting output from SAS:

Dependent Variable: Seller Earning

| Model Description | |
| --- | --- |
| Estimation Method | Parks |
| Number of Cross Sections | 6 |
| Time Series Length | 20 |

| Fit Statistics | | | |
| --- | --- | --- | --- |
| SSE | 47.4701 | DFE | 108 |
| MSE | 0.4395 | Root MSE | 0.6630 |
| R-Square | 0.9791 |  |  |

| Parameter Estimates | | | | | |
| --- | --- | --- | --- | --- | --- |
| Variable | DF | Estimate | Standard Error | t Value | Pr > \|t\| |
| No Seizure | 1 | 99.14648 | 5.2829 | 18.77 | <.0001 |
| Product Seizure | 1 | -36.5419 | 6.7333 | -5.43 | <.0001 |
| Trade Seizure | 1 | -53.1955 | 9.7282 | -5.47 | <.0001 |
| Seller Profit Seizure | 1 | -14.3297 | 6.0203 | -2.38 | 0.0191 |
| Both Profit Seizure | 1 | -8.6178 | 6.6144 | -1.30 | 0.1954 |
| Buyer Profit Seizure | 1 | -6.69552 | 6.6023 | -1.01 | 0.3128 |

## **SAS code for Buyer Earnings**

The PANEL Procedure Parks Method Estimation was conducted for Buyer Earnings in SAS. The following is the SAS code used for the variable Buyer Earnings:

“**PROC** **PANEL** DATA=TWO;

ID TREATMENT PERIOD;

MODEL BUYEARN = ASYM PLASYM TLASYM SPLASYM BPLASYM YPLASYM SL PLSL TLSL SPLSL BPLSL YPLSL / PARKS NOINT;

OUTPUT OUT = RESULT P = PREDICTED R = RESIDUAL;

**PROC** **UNIVARIATE** NORMAL PLOT;

VAR RESIDUAL;

**RUN**;”

The SAS code gave the following results:

Dependent Variable: Buyer Earnings

| Model Description | |
| --- | --- |
| Estimation Method | Parks |
| Number of Cross Sections | 6 |
| Time Series Length | 20  Fit Statistics |

| SSE | 47.5201 | DFE | 108 |
| --- | --- | --- | --- |
| MSE | 0.4400 | Root MSE | 0.6633 |
| R-Square | 0.9973 |  |  |

| Parameter Estimates | | | | | |  |
| --- | --- | --- | --- | --- | --- | --- |
| Variable | DF | Estimate | Standard Error | t Value | Pr > \|t\| | |
| No Seizure | 1 | 143.206 | 3.4488 | 41.52 | <.0001 | |
| Product Seizure | 1 | -31.065 | 4.3297 | -7.17 | <.0001 | |
| Trade Seizure | 1 | -41.9153 | 6.1958 | -6.77 | <.0001 | |
| Seller Profit Seizure | 1 | -1.29492 | 5.5344 | -0.23 | 0.8154 | |
| Both Profit Seizure | 1 | -33.2448 | 4.0345 | -8.24 | <.0001 | |
| Buyer Profit Seizure | 1 | -27.3009 | 3.4848 | -7.83 | <.0001 | |

## **SAS code for Total Earnings**

The PANEL Procedure Parks Method Estimation was conducted for Total Earnings in SAS. The following is the SAS code used for the variable Total Earnings:

“**PROC** **PANEL** DATA=TWO;

ID TREATMENT PERIOD;

MODEL TOTEARN = ASYM PLASYM TLASYM SPLASYM BPLASYM YPLASYM SL PLSL TLSL SPLSL BPLSL YPLSL / PARKS NOINT;

OUTPUT OUT = RESULT P = PREDICTED R = RESIDUAL;

**PROC** **UNIVARIATE** NORMAL PLOT;

VAR RESIDUAL;

**RUN**;”

The following is the resulting output from SAS:

Dependent Variable: Total Earnings

| Model Description | |
| --- | --- |
| Estimation Method | Parks |
| Number of Cross Sections | 6 |
| Time Series Length | 20 |

| Fit Statistics | | | |
| --- | --- | --- | --- |
| SSE | 47.9123 | DFE | 108 |
| MSE | 0.4436 | Root MSE | 0.6661 |
| R-Square | 0.9983 |  |  |

| Parameter Estimates | | | | | | |
| --- | --- | --- | --- | --- | --- | --- |
| Variable | DF | Estimate | Standard Error | t Value | Pr > \|t\| |  |
| No Seizure | 1 | 968.2817 | 20.5992 | 47.01 | <.0001 |  |
| Product Seizure | 1 | -269.714 | 33.7352 | -8.00 | <.0001 |  |
| Trade Seizure | 1 | -380.726 | 33.9765 | -11.21 | <.0001 |  |
| Seller Profit Seizure | 1 | -59.7586 | 23.6376 | -2.53 | 0.0129 |  |
| Both Profit Seizure | 1 | -166.2 | 28.4737 | -5.84 | <.0001 |  |
| Buyer Profit Seizure | 1 | -134.408 | 26.5196 | -5.07 | <.0001 |  |

## **STATA code for Wilcoxon Rank-Sum tests**

The following command was executed over only the last five periods for each of the variables of interest over all treatments:

“ranksum ‘variable’ if period>15 & treatment==X | periods>15 & treatment==X, by (treatment)”

The results are given in Table A of S2 Text.

## **STATA code for Regression Analyses**

The following STATA code was executed using the entire data reported in Table D to conduct the regression analyses reported in Table C of S2 Text:

“regress variable i.treatment percentfemale if period>15”

This code was replicated for each of the reported variables.
