# Supplementary material for: The relative effectiveness of law enforcement policies aimed at reducing illegal trade: Evidence from laboratory markets
Source: PLoS One. 2021 Nov 2;16(11):e0259254. doi: 10.1371/journal.pone.0259254 (PMC8562810; doi:10.1371/journal.pone.0259254)
Supplement: Attachment — Submitted filename: Response to Reviewers.docx [file pone.0259254.s004.docx]

Dear Dr. Miettinen,

Thank you for the opportunity to submit an improved revision of the manuscript originally titled, “Money Laundering, Crime, and the Seizure of Assets: Relative Effectiveness of Law Enforcement Policies in Experimental Bargaining Markets.” We sincerely appreciate the valuable comments provided by yourself and the two reviewers. We have attempted to address them all, and believe the manuscript is much improved because of them. Below is a detailed response to all the comments made by yourself and reviewers, specifying changes to the original manuscript to address the concerns. Changes to the manuscript are noted by blue in the text.

**Dr. Miettinen:**

**I have read the paper myself and I can only agree with their evaluation. In addition to their criticism, I would like to raise a few further points I would like to you to pay attention to in your revision**

**1) You mention in the paper that practice periods were run up to the point where no participant asked for further practice rounds. I would like you to very briefly report some treatment level statistics on the number of practice rounds as this could have an effect on treatment differences. Such variation is not a reason to reject the paper, but it is valuable information for the reader when evaluating the mechanisms behind the treatment effects.**

We agree that including treatment level statistics on the number of practices periods is important information for the reader. We have included a table including the summary statistics on the number of practice periods per session for each treatment in Table 3 on page 23. We further explain that due to the small variation in practice periods across treatments, we do not expect this to have an influence on treatment effects. We include the following to lines 497-499 on page 23 to explain this:

*“Prior to the start of the experiment, participants took part in at least one practice period. The average number of practice periods varied slightly between the treatments (Table 3), but not enough to expect a behavioral effect on treatment differences.”*

**2) The trading mechanism is repeat bilateral bargaining with an exogenously set number of matches (3). This procedure differs from the more standard practices of double auction markets. I would like you to discuss briefly, referring to existing literature if necessary, why you think the competitive equilibrium (which you use in deriving your predictions) should provide accurate predictions for the this market mechanism.**

Thank you for your comment. We use the competitive equilibrium only as a guide to understand potential policy impacts prior to testing. Our primary objective is not to test theoretical market outcomes, but to understand real-world market outcomes. Thus, we choose parallelism in our laboratory market design for illegal products and choose bilateral bargaining as the appropriate trading institution.

Davis and Holt (1993) state that in markets where agents make decisions sequentially and in real time, trading institutions are much more difficult to analyze theoretically (including double auction and bilateral negotiation), but these institutions offer parallelism and are closer to institutional rules found in many real-world markets. The double auction often is used in experimental market research, as it is thought to yield results mimicking the competitive equilibrium. Yet, research investigating differences in trading institutions and delivery methods relevant in commodity markets finds that neither the double auction nor private negotiation yields precisely the competitive equilibrium (Menkhaus et al., 2003). Menkhaus et al. (2003) find that under the same supply and demand conditions as used in the current study, the double auction with forward delivery yields prices below and trades above the predicted competitive equilibrium. The double auction with spot delivery was found to have a converged price above the competitive equilibrium and trades equal to the equilibrium. Private negotiation with forward delivery and three bargaining matches yields prices above the competitive equilibrium and trades below the predicted equilibrium. Private negotiation with spot delivery yields price and trades below the competitive equilibrium. Thus, neither institution was found to generate the competitive equilibrium perfectly.

As both Davis and Holt (1993) and Menkhaus et al. (2003) show, even though double auction is a common institution used in the literature, it also cannot be expected to yield the expected equilibrium in all cases. We do acknowledge that is has been found to produce market outcomes that are closer to the predicted equilibrium when compared to bilateral bargaining. To our knowledge, no illegal product market uses double auction and its formal trading rules. There is, therefore, a resulting trade-off between parallelism of illegal product markets and convergence to predicted outcomes with our choice of institution. For our purpose of measuring real-life market outcomes in illegal product markets, we feel that parallelism is more important.

We do agree that additional language is necessary in the manuscript to further explain our reasoning for using bilateral bargaining instead of the double auction institution. We have added the following to lines 218-223 on page 10:

*“Many market experiments use a double auction institution, as it is thought to yield results mimicking the competitive equilibrium [36]. However, the market institution through which illegal products reach the consumer is bilateral bargaining. Given this reality, past research has used a bilateral bargaining market for illegal product sales (e.g. [37]). Since our intent is to understand market outcomes in real-world illegal product markets, we use bilateral bargaining, as it offers closer parallelism than the double auction to these markets [37].”*

References:

Davis, D. D., and C. A. Holt. 1993. “Chapter 1: Introduction and Overview.” *Experimental Economics*. Princeton, New Jersey: Princeton University Press. Pp. 3-66.

Menkhaus, D. J., O. R. Phillips, and C. T. Bastian. 2003. “Impacts of Alternative Trading Institutions and Methods of Delivery on Laboratory Market Outcomes.” American Journal of Agricultural Economics. Vol 85. No. 5: 1323-1329.

**3) The competitive equilibrium prices and quantities depend on the slopes and intercepts of the demand and supply schedules. I would like to better understand, how your treatments are hypothesized to effect on these slopes and intercepts, e.g. on the expected marginal costs of the seller. A figure depicting these schedules in each treatment might be useful, maybe condensed into one figure or a figure with several panes to allow for easy comparison of the predictions.**

We agree that a figure including the schedules and predicted equilibria improves the paper. We have included Figure 1 on page 17 that depicts the effective supply and demand schedules along with the predicted equilibria by using panes for each treatment.

**When submitting your revision, we need you to address these additional requirements.**

**1) Please ensure that your manuscript meets PLOS ONE's style requirements, including those for file naming.**

We have changed file names that now meet the style requirements.

**2) Please consider changing the title so as to meet our title format requirement. In particular, the title should be "Specific, descriptive, concise, and comprehensible to readers outside the field" and in this case it is not informative and specific about your study's scope (in particular the 'lab' setting) and methodology.**

We have changed the title of the manuscript to:

*“The Relative Effectiveness of Law Enforcement Policies Aimed at Reducing Illegal Trade: Evidence from Laboratory Markets”*

**3) Please include captions for *all* your Supporting Information files at the end of your manuscript, and update any in-text citations to match accordingly.**

We now include captions for each of the three Supporting Information files at the end of the edited manuscript.

**Reviewer #1:**

**This paper aims to compare the effectiveness in trade reduction of three enforcement policies: profit seizure of either buyers, sellers or both, product seizure after production and product seizure after a trade is negotiated. To address this question, the authors derived equilibrium predictions and designed a market experiment to test their main comparative statics in which they manipulate the enforcement institution.

The authors find that profit seizure policies do not reduce the volume of quantities produced or traded compared to the volume in the absence of regulation. In contrast, product seizure after trade has the greater impact on both quantity produced, and quantity traded.

The authors contribute to the literature by enhancing our understanding of where in the production-trade-laundering cycle, enforcement policies are the most effective at curbing illegal trade. The experimental design enables clean comparisons between the three policies in terms of quantities produced and traded, prices and profits.**

Thank you

**Nonetheless, the paper would benefit from some clarifications.

1. From the introduction, the reader is led to believe that most enforcement policies aiming at curbing illegal trade currently in place are targeting the money laundering stage of the cycle. However, there are often reports in the news of drugs seizure after trade were negotiated (e.g. during transport) or after production. From what I understand, the problem is that enforcement currently takes place anywhere along the production-trade-laundering cycle but there is no evidence yet on where along this cycle enforcement is the most efficient. If this is the case, I would advise the authors to reorganize their introduction along these lines and to not overextend on policies aiming at reducing money laundering only. If it appears that most of the effort is concentrated towards the laundering stage of the cycle, then it poses the question of why it is the case: is it because it is more difficult to implement policies at earlier stages of the cycle? A discussion on these aspects seems highly relevant to appreciate the policy implications of the paper.**

Thank you for this very meaningful comment. Our intent was to show that although there are various law enforcement policies along the production-trade-laundering cycle, currently, there is a push for policies to focus on the money-laundering stage. We have added and edited text in the abstract and the introduction to better reflect our intention. Specifically, the abstract now includes the following:

*“Despite recent emphasis and implementation of national and international anti-money laundering policies, illegal product markets, and their associated illicit profit remain a global problem. In addition to law enforcement aimed at reducing money-laundering, enforcement also takes place during (1) the production (e.g. crop eradication) and (2) sale (e.g. seizure of products during transportation that interrupts buyer and seller transactions) of the illegal product.”*

The introduction has been expanded and now includes the following to lines 58-81 on pages 3-4:

*“Globalization has provided new opportunities for the proliferation of illegal product distribution. As a result, many international policies exist to halt the production and distribution of illegal products, and the laundering of illicit profit. These policies mainly use the seizure of assets throughout the production-trade-laundering cycle of illegal product markets to attack the financial structure and money laundering of groups worldwide [4]. Other penalties, including fines and prison terms, can be conceptualized as a seizure of assets from a broader perspective. Asset seizure and forfeiture is popular because it can reduce trade activity and profits (and therefore punish) of all entities involved, even though it may be difficult to identify all involved parties [5]. In 2020, nearly $600 million worth of illegal drugs and related assets were seized in the United States alone [4].*

*Various policies focus on seizing the production of illegal products before they are sold to consumers. For instance, the Drug Enforcement Administration (DEA) uses crop eradication to reduce the quantity of illegal drugs before they can hit the market. In 2019, the DEA eradicated over 4 million marijuana plants and seized nearly $30 million dollars of cultivator assets [6].*

*In addition to policies that eradicate illegal products to reduce the amount of product on the market, policies also aim to capture assets during the sale of these illegal products. These policies include street-level enforcement to interrupt transactions and border enforcement [7]. Border enforcement focuses on seizing illegal products as they cross international boundaries. Since most of these products are in transit to their buyer, seizure at the border, like street-level enforcement, aims law enforcement actions during the sale or trade of illegal products.*

*Although law enforcement polices exist that focus on the production or sale of illegal products, recent emphasis has been placed on heightened enforcement aimed at the laundering of illicit profit.”*

We have also added the following to lines 90-91on page 4:

*“As a result of the recent spotlight on money laundering, numerous national and international anti-money laundering policies and entities have been created over the last 40 years.”*

We have also added the following to lines 117-120 on page 6:

*“Since funds for enforcement come from limited budgets, focusing enforcement in one stage of this cycle, likely results in less enforcement available for the other stages [15]. Thus, understanding where in this production-trade-laundering cycle law enforcement is most impactful becomes a global question.”*

**2. I cannot understand how to read the letters in Table 3 that denote the significance of pairwise comparisons between treatments. Perhaps there is a more intuitive way to do this? Otherwise, perhaps the authors could clarify how these letters should be interpreted.**

Thank you for highlighting the confusion of the letters used in the table. We have edited the table to now only include statistical differences of the treatments to the base (No Seizure treatment). We now only include * to indicate a significant treatment effect with respect to the No Seizure treatment. We think this change addresses the confusion you indicate and does not lose any vital information.

**3. Regarding Table 3 again, the authors indicate significance at the 10% level. Given the strong significance of the results, I would recommend the authors to be more conservative and only indicate significance at the 5% level. Doing so will not affect their main results and reassure the reader.**

This is a great point, and we have updated Table 4 to only indicate significant treatment effects at the 5% level.

**Reviewer #2:**

**This paper examines the effect of law enforcement on outcomes in "illegal" markets using an lab experiment. It concludes that a policy based on seizure of profits has little effect but targeting the point of sale is effective.**

**1) The characteristics of the participants in each treatment need to be presented and tests of equality/balance carried out.**

Every attempt was made to randomize participants across the treatment. We do recognize that with experiments such as ours, our attempts may still not lead to full randomization of participants over the treatments. Based on a previous study showing that gender may impact individual outcomes in these types of laboratory market experiments, we collected the gender of participants. We have now added the proportion of women per session across the treatments in Table 3 on page 23.

We have also included a test of balance of participants’ gender across treatments using the method outlined by Imbens and Rubin (2015), which is included in S2 Text. We do find that gender is imbalanced across the treatments. We have added statistics on the proportion of women per session in Table 3 on page 23, and added the following text in the main manuscript to lines 499-504 on page 23 to state our finding:

*“Participants’ gender was also collected since previous literature shows that it can affect individual outcomes in similar market experiments [41]. Every attempt was made to fully randomize participants across the treatments. Yet, based on the method outlined by Imbens and Rubin [51], our sample is imbalanced in respect to gender. As a result, gender (defined as the proportion of women participants in a session) is included as a covariate in regression analyses that are compared to the convergence analysis results (Table B in S2 Text).”*

Unfortunately, we did not collect information on other characteristics of the sample. We think this is an important avenue for future research.

**2) Seizure as a policy in the "real world" entails costs over and above the amount seized. As law enforcement detects illegal activity and the channels through which the profits are laundered, criminals must hire new agents or create new ways to launder their money. There is also a chance that they will lose all profits and exit the game entirely. I think this needs to be acknowledged as seizure of profits in the experiment simply sees the "criminals" lose tokens with some probability.**

We agree that we did not highlight that in the real world, there are other costs beyond the loss of laundered profit. To address this issue, we have added the following to lines 343-345 on page 16:

*“When law enforcement seizes laundered money, for example, criminals must find new avenues to launder their illicit profit, which can be very costly.”*

To note that we are modeling large entities in our market, and that smaller agents may lose all product or profit from law enforcement action, we have added the following to the Conclusion section on page 32 to lines 694-703:

*“Further, the buyers and sellers modeled in this study are assumed to represent large entities where law enforcement, even when successful, cannot seize all illegal assets at any stage along the production-trade-laundering cycle. It is thus assumed that operations modeled are large enough, for instance, that if one field of illegal crop is eradicated, there are many other fields left undetected, or illicit profit is laundered through multiple avenues, such that, even with detection of money laundering in some avenues, other avenues remain undetected. Yet, the potential exists for law enforcement to successfully seize all assets of smaller operations than assumed here. Additionally, our analysis does not capture the ability of operations to reinvest profits into capital acquisition to expand production capabilities or invest in other criminal activities. Future research should focus on how effective various law enforcement policies are in such situations.”*

**3) Are the participants equally distributed across the treatments?**

Yes, participants were equally distributed across the treatments, i.e., the same number of participants were used for each experimental session, and different subjects were used for each session. We have added the following text to page 13, lines 274-276 of the main manuscript:

*“We use a between-subjects design where subjects were recruited to participate in only one session and treatment. Subjects selected a session to participate in, after which, the treatment was randomly assigned to each session. Each session had eight participants.”*

Further, every attempt was made to randomize participants across the treatments. Yet, as stated prior, we do recognize that our attempts may still not lead to full randomization of participants across treatments. We do find that the sample is imbalanced in respect to participant gender and conduct analyses controlling for gender to ensure our results are not biased by such imbalance.

**4) I am unfamiliar with the parametric convergence model and I think the rationale for using it should be discussed. In any event, for transparency and completeness I would like to see the analysis starting with a simpler method of comparing means across treatments and running regressions with treatment effects (including any personal characteristics that vary by treatment).**

Thank you for your comment. We have added the following text to pages 21-22, lines 465-472 to more fully explain the rationalization for using the convergence analysis:

*“The convergence model provides a statistical description of the path of the data from the beginning level to the asymptote of each treatment while addressing econometric issues found in the experimental data (such as heteroskedasticity, contemporaneous correlation, and serial correlation) [46]. As t increases, the weight of the starting value becomes smaller, while the weight of the asymptote becomes larger. Through this analysis, we can evaluate treatment effects by testing for differences in the estimated asymptotes for each treatment without making a priori judgements on the appropriate data that represents stable convergence, such as is needed in more simplistic difference of means tests or other tests that do not account for t.”*

To add validity to the convergence analysis results, we have also included results from simultaneous difference of means tests (through Tukey tests) in Table A in S2 Text. Further since gender has been previously found to influence outcomes in such market experiments and is found to be imbalanced across the treatments, we also report regression analyses that include the percent of women in a session as a covariate in Table B in S2 Text. We have added the following to the main manuscript to explain the inclusion of the Tukey test and regression analyses to pages 22 and 23, lines 488-494:

*“To lend validity to the convergence analysis, results are compared to simultaneous difference of means two-tailed t-tests via Tukey tests and regression analyses over the last five periods (which is used in the previous literature to represent convergence [30-31,50]) when including covariates found to potentially influence market outcomes from previous literature (S2 Text). Although including covariates when measuring treatment effect leads to biased estimators [51], recent literature suggests that properly adjusting for any imbalanced prognostic variables is appropriate [52-53]. These additional analyses are performed via STATA statistical software.”*

We find the results from the Tukey and regression analyses are consistent with the results from the convergence analysis. We have noted the consistency between the results in the manuscript. Specifically, on page 24, lines 520-523, we state:

*“These results are consistent with difference of means Tukey tests (Table A in S2 Text) and the regression analysis (the only difference to note is that the regression analysis found that the Both Profit Seizure treatment had significantly more units produced than the No Seizure treatment; Table C in S2 Text).”*

On page 25 lines 541-543, we state*:*

*“Again, these results are consistent with the regression analysis and difference of means test (except that the Tukey test did not find a significant treatment effect for the Both Seizure treatment; Table A and C in S2 Text).”*

On page 27, lines 578-580, we now state:

*“These results are supported by the regression and Tukey results, lending further support to these outcomes (Table A and C in S2 Text).”*

We also note that results are, *“consistent with regression and Tukey results; Table A and C in S2 Text”* twice on page *27.*
