# Supplementary material for: The relative effectiveness of law enforcement policies aimed at reducing illegal trade: Evidence from laboratory markets
Source: PLoS One. 2021 Nov 2;16(11):e0259254. doi: 10.1371/journal.pone.0259254 (PMC8562810; doi:10.1371/journal.pone.0259254)
Supplement: Attachment — Submitted filename: Response to Reviewers.docx [file pone.0259254.s005.docx]

Dear Dr. Miettinen,

Thank you for the opportunity to submit a second improved revision of the manuscript, “The Relative Effectiveness of Law Enforcement Policies Aimed at Reducing Illegal Trade: Evidence from Laboratory Markets.” We sincerely appreciate the additional comments provided by yourself and the two reviewers. We have attempted to address them all in the manuscript and in this response. We believe the manuscript is much improved because of these constructive comments. Below is a detailed response to the comments made by yourself and two reviewers, specifying changes to the original manuscript to address the concerns. Changes to the manuscript are noted by blue in the text.

Dr. Miettinen: **Reviewer #2 is more critical and he has concerns about (i) randomization into treatments, (ii) balance, and (iii) statistical analysis. I agree with her/him that randomization is not perfect -- ideally each participant would have an i.i.d. chance of ending up in each treatment and this is something the experimental economics community should pay more attention to. You do not have many participant characteristics to judge your balance against but gender is suggestive of some, but maybe not all that dramatic, failure in this respect. You are very open about this challenge in the paper, so it is easy for the reader to discount when judging the evidence.**

**Reviewer #2 is also critical about the statistical analysis and calls for panel regressions with data from all rounds. While I agree with that being a valid approach, I also see value in treating each session as an independent observation and looking at long run "equilibrium" effects. This approach has been used in the literature as you mention. The value of this approach is slightly downplayed by the fact that your theoretical predictions are suggestive at best. I would like you to add one more robustness check as I think the normality assumption in the Tukey tests could be a bit heroic: I'd like you to report Wilcoxon rank-sum tests (they can be one sided as you are clearly expecting a reduction in quantities etc.) session averages in the last five periods being the independent observations.**

We agree that the normality assumption used by the Tukey tests may be heroic. We have replaced the Tukey tests with Wilcoxon rank-sum tests over the last five periods and report them in S2 Text. The results from the Wilcoxon rank-sum tests are consistent with the convergence and regression analyses. We reference the Wilcoxon’s non-parametric Rank-Sum tests throughout the revised manuscript.

Further, in our response to Reviewer 2, we provide additional literature to support and clarify our approach and report further analyses to show our results are robust. Since our focus in the manuscript is on equilibrium outcomes, we only report these additional results in this response, and not in the manuscript itself (or supplemental appendices).

Reviewer #1: **The authors put a lot of effort in addressing my comments from the previous round of reviews, which is much appreciated.**

Thank you

**The introduction is now clearer about the contribution of the paper. However, I still believe the exposure can be improved:

In its current version, the introduction describes some empirical examples of seizure policies from lines 67 to 78. It then goes on about the recent interest on money laundering until line 112. Then the main argument of the paper is outline from line 113 to 123. This structure is not only a bit redundant, but also not straightforward.

A more natural flow would be to state the most important paragraph (from line 113 to 123) upfront. Then described the three policies while integrating the empirical examples (instead of keeping them separate, then making the point about money laundering policies and concluded on the fact that while policy makers focus on this one particularly, there is no evidence that it is where the seizure is most efficient. I would then keep the paragraph from line 124 to 135, as it is a nice transition to the rest of the paper.**

Thank you for this very insightful comment. We have reorganized the introduction so that anti-money laundering policy is introduced earlier. This reorganization also integrates the empirical examples of the various policies, and then focuses on the current prevalence of anti-money laundering policies. Specifically, lines 66-111 on pages 3-5 now reads:

*“Although law enforcement polices exist that focus on the production or sale of illegal products, recent emphasis has been placed on heightened enforcement aimed at the laundering of illicit profit. Generally, illegal trading relies on cash transactions, where the cash must be disguised to avoid legal entanglements. This cash is frequently passed through banks and other financial institutions in order to make its source difficult to trace [6]. This form of money laundering disguises the source of proceeds from criminal activity by making it appear as if earnings are legitimate. The amount of cash flowing through illegal transactions is estimated to account for two to five percent of global GDP yearly [6]. In addition to potential societal harm from illegal trade activity, money laundering and the injection of laundered funds into new ventures create economic damage. It is estimated that an increase of USD 1 billion in money laundering can reduce economic growth between 0.03 and 0.06 percentage points [7].*

*Various other policies focus on other stages throughout the production-trade-laundering cycle. For instance, the Drug Enforcement Administration (DEA) uses crop eradication to reduce the quantity of illegal drugs before they can hit the market. In 2019, the DEA eradicated over 4 million marijuana plants and seized nearly $30 million dollars of cultivator assets [8]. Additionally, other policies include street-level enforcement to interrupt transactions and border enforcement [9] to capture assets during the sale of the illegal products. Border enforcement focuses on seizing illegal products as they cross international boundaries. Since most of these products are in transit to their buyer, seizure at the border, like street-level enforcement, aims law enforcement actions during the sale or trade of illegal products. Although these policies aimed at the other stages in the production-trade-laundering cycle can be impactful, anti-money laundering policies have been the focus of recent anti-crime campaigns.*

*Numerous national and international anti-money laundering policies and entities have been created over the last 40 years. Examples of such entities include the Financial Action Task Force, the Global Programme Against Money Laundering, and The International Convention Against Transnational Organized Crime, initiated through global organizations such as the United Nations and International Monetary Fund (see [10] for a review). Although increases in anti-money laundering enforcement from these programs is projected to decrease crime [11], many studies find that the effectiveness of these initiatives is limited (e.g. [12]). For instance, Ferwerda et al. [13] find that the Financial Action Task Force’s (an independent inter-governmental body that develops and promotes policies to protect the global financial system against money laundering) current method of blacklisting countries for money-laundering (used as an attempt to reduce money laundering) may not prevent money laundering, and may further reduce the quality of national statistics on money laundering. Further, Deleanu [12] finds evidence that current policy does not incentive accurate reporting by countries of money laundering, supporting the notion that national statistics on money-laundering activity are not accurate, and may even be misleading.*

*In another instance of how current anti-money laundering policies may have limited effectiveness, Takáts [14] finds that current polices requiring banking institutions to identify and report suspicion of money laundering may backfire. The Suspicious Activity Report, introduced in 1996 by the Financial Crime Enforcement Network for reporting of any suspicious activity, is one such policy. To incentivize compliance, banks and other financial institutions are charged a fee for not reporting instances of money-laundering. Current increases in this fee can cause institutions to over-report potential instances of money-laundering, creating a ‘cry wolf’ phenomenon, which reduces the efficacy of the program to reduce money-laundering, and thus crime [14].”*

**Whether the above suggestions are implemented by the authors should not affect the final decision, as in my opinion the paper is acceptable in its current version. I am merely giving the authors one last opportunity to deliver the best possible version of their work.

I am globally satisfied with the proposed changes, and I believe the paper can be accepted for publication in its current version.**

Thank you

Reviewer #2: **The authors have addressed many of my concerns but I still have reservations about the balance of the sample and the regression analysis. It is not clear to me why only 5 periods would be used (from the 20+ available). My understanding is that the model is estimated on the averaged values for those 5 rounds.**

Thank you for your comments. The convergence regression is based on all of the market data for 20 periods (to have balanced panel data) for each treatment, not just five periods (except for the variable, *Units Produced*, since it is not normally distributed, and a nonparametric analysis is conducted). The model does put less weight on earlier periods than later periods to account for learning. Thank you for highlighting this source of confusion. We have now added the following text to the footnote for Table 3 on lines 519-522:

*“Units Produced do not meet normality and are severely skewed as per Brown [49]. Thus, following previous research, we report averages for the last 5 trading periods per treatment and non-parametric test results [30-31,50]. All other variables meet the normality assumption and the convergence results and parametric tests are reported using data from all 20 trading periods”.*

Using the later trading periods for testing treatment effects (for variables that are not normally distributed) is a standard practice in the literature for market experiments focusing on equilibrium outcomes (e.g., Sabasi et al., 2013, Phillips et al., 2003, Davis and Holt, 1993, Mestelman and Welland, 1988).

We only use the last five periods in the supplemental analyses since variability from learning across subjects and treatments could impact the ability of parameter estimates to capture the magnitude of treatment effects after learning.

Noussair CN, Plott CR, Riezman RG. 1995. “An experimental investigation of the patterns of international trade.” *American Economic Review* 85(3):462-91.

Mestelman, S., and D. Welland. 1988. Advance production in experimental markets.” *Review of Economic Studies*. 55,4: 641-654.

Davis, D. D. and C. A. Holt. 1993. “Chapter 9 Economic Behavior and Experimental Methods: Summary and Extensions.” In *Experimental Economics*. Princeton University Press, Princeton, NJ.

Phillips, O. R., D. J. Menkhaus, and K. T. Coatney. 2003. “Collusive Practices in Repeated English Auctions: Experimental Evidence on Bidding Rings,” *American Economic Review*, 93,3: 965-979.

Sabasi, D. M., C. T. Bastian, D. J. Menkhaus, and O. R. Phillips. 2013. “Committed Procurement in Privately Negotiated Markets: Evidence from Laboratory Markets,” *American Journal of Agricultural Economics*, 95,5:1122-1135.

**Typically, these kinds of models would be estimated on the full panel data with period fixed effects to account for learning (and perhaps a dummy to capture if it were a "practice" round).**

We do agree that various econometric approaches are used in the literature. Yet, in laboratory market studies focusing on equilibrium outcomes (not individual outcomes), the convergence analysis is an established technique (see for example Plott and Pogorelskiy, 2017, and the other references noted in the manuscript). The benefit of using the convergence model regression method (Noussair et al. 1995) instead of fixed effects regression analysis, is that the convergence analysis addresses all the panel data characteristics that could impact econometric efficiency issues. Further, the convergence analysis more accurately follows the path of the data to test for differences in asymptotes, which are the statistically predicted equilibrium outcomes from the data for each experimental treatment.

We have conducted a period fixed effects regression below (using the full panel data) to provide further validity to the results from the convergence analysis. We do find more statistically significant results than from either the convergence analysis or the regression analysis only including the last five periods. We note differences in yellow (see Table below). These differences only further highlight our general conclusion that anti-money laundering policies are likely ineffective at reducing crime (unlike in the convergence and regression analyses on only the last five periods, we find a significant *increase* in trades in the Seller Profit Seizure treatment when compared to the No Seizure treatment: see Table below). The results also further support our other key finding that only when law enforcement is aimed at trade is crime likely reduced (only in the Trade Seizure treatment are both Units Produced and Units Traded significantly lower than in the No Seizure treatment).

|  | **Dependent Variable** | | | | | |
| --- | --- | --- | --- | --- | --- | --- |
| **Independent Variable**  (No Seizure is the Omitted treatment) | **Units Produced** | **Units Traded** | **Price** | **Seller Earnings** | **Buyer Earnings** | **Total Earnings** |
|  |  |  |  |  |  |  |
| Seller Profit Seizure | 0.437 | 0.813* | -2.917* | -19.92* | 7.647* | -49.38* |
|  |  |  |  |  |  |  |
| Buyer Profit Seizure | -0.139 | -0.115 | -1.709 | -6.401 | -25.26* | -126.80* |
|  |  |  |  |  |  |  |
| Both Profit Seizure | 1.518* | 1.917* | 2.550* | -2.11 | -30.87* | -132.10* |
|  |  |  |  |  |  |  |
| Product Seizure | 1.268* | -1.59* | 4.856* | 32.71* | -28.70* | -245.80* |
|  |  |  |  |  |  |  |
| Trade Seizure | -1.952* | -1.650* | -2.463* | -58.88* | -42.68* | -406.4* |
|  |  |  |  |  |  |  |
| Percent Women (in Session) | 2.690* | 3.208* | 12.07* | 52.13* | -8.39 | 191.2* |
|  |  |  |  |  |  |  |
| Constant | 14.19* | 13.05* | 68.48 | 68.71* | 149.00* | 870.90* |
| F-stat | 20.09 | 35.06 | 46.45 | 35.06 | 46.45 | 120.54 |

We do not report these results in the manuscript since the model’s inability to account for characteristics that could impact econometric efficiency issues potentially suggests statistical differences when none truly exist. Moreover, this period fixed effects model could be underspecified and have biased parameter estimates. Thus, to limit the risk of Type I errors, the convergence analysis is reported in the manuscript.

We also have conducted an alternative Time Series Cross Sectional Model which includes treatment dummies, a trend, and a fixed effects dummy for learning using all 20 periods of data and estimated with the PARKS method panel data estimator in SAS (see Table below). This model is more appropriate than the fixed effects regression since it accounts for some of the characteristics in the data that can affect econometric efficiency. It also is better specified in that it addresses potential trends in the data over time. The results of this model are also similar to the convergence model results, and again lends further support to the findings reported in the manuscript. The R-squares are lower in this model since it does not follow the path of the data (i.e., predict the dependent variable as well) and our estimates of converged values at equilibrium from this model are different than the convergence model in several instances given the concerns previously expressed regarding this approach.

|  | **Dependent Variable** | | | | | |
| --- | --- | --- | --- | --- | --- | --- |
| **Independent Variable** | **Units Produced** | **Units Traded** | **Price** | **Seller Earnings** | **Buyer Earnings** | **Total Earnings** |
|  |  |  |  |  |  |  |
| Seller Profit Seizure | 0.312 | 0.867* | -4.194* | -22.861* | 8.812 | -62.729* |
|  |  |  |  |  |  |  |
| Buyer Profit Seizure | -0.328 | 0.087 | -4.278* | -13.589 | -25.040* | -155.836* |
|  |  |  |  |  |  |  |
| Both Profit Seizure | 1.204* | 2.068* | 0.126 | -8.116 | -30.032* | -158.433* |
|  |  |  |  |  |  |  |
| Product Seizure | 1.147* | -1.422* | 3.634* | -36.579* | -28.451* | -258.920* |
|  |  |  |  |  |  |  |
| Trade Seizure | -1.905* | -4.680* | -2.111* | -57.771* | -42.804* | -402.138* |
|  |  |  |  |  |  |  |
| Percent Women (in Session) | 0.064 | 0.418* | -0.32 | 0.933 | -0.548 | 1.724 |
|  |  |  |  |  |  |  |
| Learn Dummy | 0.535* | 0.114 | 1.54 | -1.634 | 0.118 | -4.181 |
| Trend | -0.010 | -0.234* | 0.497* | 1.054 | -0.949 | 0.708 |
| Constant | 15.956* | 14.875* | 70.134* | 79.436* | 159.542* | 952.074* |
| R-Square | 0.8608 | 0.8492 | 0.8441 | 0.8342 | 0.9133 | 0.9125 |

These results also support the conclusion in the manuscript that the amount of illegal product available and traded in the market is reduced most when seizing units during trade. Further, the conclusion that illicit trade is either increased or not statistically different from the base treatment when enforcement is aimed at money laundering is again consistent with the results presented in the manuscript.

The practice rounds used different unit costs for sellers and different redemption values for buyers (which is common practice to not have participants establish expectations for the actual experiment). Any regression analyses using the practice rounds would be unfitting as they would have different predicted equilibrium values than the actual data.

Plott, C. R., and K. Pogorelskiy. 2017. “Call Market Experiments: Efficiency and Price Discovery through Multiple and Emergent Newton Adjustments,” *American Economic Journal: Microeconomics* 9,4: 1-41.

**Given how unbalanced the sample was in terms of gender, one worries that other unobserved/unmeasured characteristics could be driving the results.**

The market experiments used in this study rely on induced value theory (Smith, 1976) where “individuals’ innate characteristics become largely irrelevant” (Friedman and Sunder, 1994, p.13). Moreover, as we are interested in market equilibrium effects of policy, we run six replications to further reduce the potential impact of individual impacts. Since we use multiple trading periods, with three bargaining rounds each, across multiple replications, and random assignment of treatment and agent role, the expectation is that subject pool/sample effects are generally reduced or washed away.

A possible solution to address the potential that unobserved characteristics are driving the results is to have used a different sample choice. As we do not know the population characteristics of those involved in illegal product markets, we cannot draw a sample representative of the actual population involved in such markets.

Further, Frechette (2015) reviews the experimental literature to investigate the use of representative or demographically varied samples, and finds, “As a whole, there are a few patterns that seem to emerge from the use of demographically varied samples. One is that results are often not drastically different from those using the standard sample of student subjects, certainly when it comes to comparative statics, and to the extent that there are differences, these can often be traced to age (over 55)” (p. 460). We do not expect our sample to vary much by age because the community where the experiment was conducted has a very young average age given the presence of the University.

Smith, V. L. (1976). “Experimental Economics: Induced Value Theory,” American Economic Review. 66: 274-279.

Friedman, D. and S. Sunder. 1994. “Chapter 2 Principles of Economic Experiments.” In Experimental Methods: A Primer for Economists. Cambridge University Press, New York, NY.

Fréchette GR. Experimental economics across subject populations. In: Kagel JH, Roth AE, editors. The handbook of experimental economics, volume 2. Princeton: Princeton University Press; 2015, pp 435-480.

**The fact that subjects "selected" a session to attend also speaks to the fear that different types of people ended up in different treatments in a non-random way.**

Participants did sign up for a time as you suggest, but the treatment to be run was chosen without the participant’s knowledge. Further, participants were randomly chosen by the computer to be either a buyer or seller in that session after logging in.

**Ultimately, we just do not know enough about the composition of the sample to have confidence in the results.**

We hope these comments and further analyses address your concerns regarding our analyses, results, and conclusions as it relates to our sample of participants.
