# Supplementary material for: The relative effectiveness of law enforcement policies aimed at reducing illegal trade: Evidence from laboratory markets
Source: PLoS One. 2021 Nov 2;16(11):e0259254. doi: 10.1371/journal.pone.0259254 (PMC8562810; doi:10.1371/journal.pone.0259254)
Supplement: Attachment — Submitted filename: Response to Reviewers.docx [file pone.0259254.s006.docx]

Dear Dr. Miettinen,

Thank you for the opportunity to resubmit the manuscript titled, “Money Laundering, Crime, and the Seizure of Assets: Relative Effectiveness of Law Enforcement Policies in Experimental Bargaining Markets,” to address your comments. Our response to each of your excellent comments are detailed below:

**1. That seller profits fall short of buyer profits is indicative of potential implications regarding the incidence of deterrence, simlar to that in the case of tax evasion. Are you in a position of saying something interesting about that and relating to existing literature? It seems in each case, you might be able to calculate the expected direct monetary consequences of seizure on sellers and then calculate to which extent the sellers can pass the cost onto buyers as higher prices. This can be very brief if you prefer, some summary of data on suggestive of a future line of inquiry. I may be mistaken here, in which case explain in your response why you are unable to do this.**

Thank you for this useful comment. We now include a table and discussion on the incidence of deterrence to both buyers and sellers on Lines 600-642:

*The increased bargaining power of buyers can be further seen by the amount of the cost of enforcement absorbed by sellers compared to buyers. Based on the theoretical predications presented in Fig 1 and Table 2, the incidence of enforcement (the percent decrease in Total Earnings from enforcement absorbed by the relevant party) for both sellers and buyers are shown in Table 5. Based on theory, sellers should only have a higher enforcement incidence than buyers when enforcement is aimed at money laundering of sellers only (Table 5). Yet, the experimental results show that sellers additionally absorb more enforcement cost than buyers in the Product Seizure and Trade Seizure treatments (the percent decrease in Total Earnings absorbed by the relevant group from Table 4 and shown in Table 5). Further, comparing the predicted seller enforcement incidence to the realized (from the experimental data) seller enforcement incidence in Table 5, sellers have a higher enforcement incidence than expected in the Buyer Seizure, Product Seizure, and Trade Seizure treatments. Thus, buyers are able to use their increased bargaining power in these treatments to push some of their cost of enforcement onto sellers.*

Table 5: Enforcement Incidence by Treatment (in Percent)

|  | **Treatment** | | | | |
| --- | --- | --- | --- | --- | --- |
| **Variable** | **Seller Profit Seizure** | **Buyer Profit Seizure** | **Both Profit Seizure** | **Product Seizure** | **Trade Seizure** |
| **Predicted Seller Enforcement Incidence ^i^** | 100 | 0 | 50 | 28 | 26 |
| **Predicted Buyer Enforcement Incidence ^ii^** | 0 | 100 | 0 | 72 | 74 |
| **Realized Seller Enforcement Incidence ^iii^** | 92 | 20 | 21 | 54 | 56 |
| **Realized Buyer Enforcement Incidence ^iv^** | 8 | 80 | 79 | 46 | 44 |
| **Difference between Expected and Realized Incidence ^v^** | 8 | -20 | 29 | -26 | -30 |

*^i^ Calculated as the expected loss in Seller Earning, divided by the expected loss in Total Earning (Seller and Buyer Earnings) in the given treatment compared to the No Seizure treatment. Values are based on the theoretical predictions presented in Table 2.*

*^ii^ Calculated as the expected loss in Buyer Earning, divided by the expected loss in Total Earning (Seller and Buyer Earnings) in the given treatment compared to the No Seizure treatment. Values are based on the theoretical predictions presented in Table 2.*

*^iii^ Calculated as the realized loss in Seller Earning, divided by the realized loss in Total Earning (Seller and Buyer Earnings) in the given treatment compared to the No Seizure treatment. Values are based on the convergence analysis presented in Table 4.*

*^iv^ Calculated as the realized loss in Buyer Earning, divided by the realized loss in Total Earning (Seller and Buyer Earnings) in the given treatment compared to the No Seizure treatment. Values are based on the convergence analysis presented in Table 4.*

*^v^ Calculated as the difference between expected and realized incidence for sellers for the given treatment. A positive value indicates a reduction in realized incidence, compared to theoretical predictions, for sellers (a benefit to sellers). A negative value indicates a reduction in realized incidence, compared to theoretical predictions, for buyers (a benefit to buyers).*

*It is only in the Seller Profit Seizure and Both Profit Seizure treatments that sellers are able to pass on some of their expected loss in earnings to buyers (Table 5). But in neither of these treatments were sellers able to pass on enough of the cost of enforcement for sellers to have higher earnings than buyers (Table 4). As such, buyers seem to have a bargaining advantage over sellers in illegal product markets.*

*Table 5 also shows that theoretical predications of incidence, which depend solely on the relative elasticity of supply and demand [57], may fail to hold for enforcement incidence in bilateral bargaining markets. This result is similar to other experimental studies of incidence (e.g. [33]), especially in markets with bilateral bargaining [31-32], since trading institution has been shown to impact incidence [58].*

We have also added the following references:

Ruffle BJ. Tax and subsidy incidence equivalence theories: experimental evidence from competitive markets. J Public Econ 2005;89:1519-42.

Cox JC, Rider M, Sen A. Tax incidence: Do institutions matter? An experimental study. Public Finance Rev 2017;46(6):899-925.

Unfortunately, due to the structure of our experiment, we do not feel we can relate our results directly to the tax evasion literature. Many of these experiments, such as those you have conducted, allow participants to make a decision to evade taxes. In real world illegal markets, participants may expend efforts to not get caught or perhaps find themselves in a position to bargain for others to get caught, such as in a prisoner’s dilemma situation. However, as the probability of seizure is exogenous in our experiment, we do not have this element of choice in our experiment. Thus, the incentives placed on participants, we feel, may cause different behavior in our study compared to the tax evasion literature. Yet, we feel this is an important element, and hope it will lead to future work in this area.

**2. The following recent publication seems related in that it attempts to address the question where deterrence is most effective. Are there other similar contributions which you could briefly relate to? Banerjee, R., Boly, A., & Gillanders, R. (2020). Anti-Tax Evasion, Anti-Corruption and Public Good Provision: An Experimental Analysis of Policy Spillovers. *Available at SSRN*.**

Again, thank you for this note. We now include the following to Lines 583-587 to state that our findings are similar to studies on enforcement on other types of crimes that find a spillover effect:

*When law enforcement seizes products during trade, there may be a spillover effect with sellers potentially leaving the market in the long run, which may further reduce illegal market activity and crime. This result is similar to other experimental studies that show effective enforcement focused on other sources of crime (e.g. tax evasion) have spillover effects that may further help in reducing crime [54-56].*

We have also added the following references:

Banerjee R, Boly A, Gillanders R. Anti-tax evasion, anti-corruption and public good provision: An experimental analysis of policy spillovers. SSRN. 2020; Available from: [http://dx.doi.org/10.2139/ssrn.3652411](https://dx.doi.org/10.2139/ssrn.3652411).

Boly A, Gillanders R, Miettinen T. Deterrence, contagion, and legitimacy in anticorruption policy making: an experimental analysis. J Leg Stud 2019;48:275-305.

Braga AA, Apel RJ, Welsh BC. The spillover effects of focused deterrence on gang violence. Eval Rev 2013;37:314–342.

**3. Please, check your list of references carefully. For instance, Fismen & Miguel -> Fisman & Miguel**

Thank you for pointing out the errors in our references. We have corrected these errors.
